# Using human-centered design to re-vision the emergency obstetric and newborn care framework: Insights from Bangladesh, Malawi and Senegal

**DOI:** 10.1371/journal.pgph.0004771

**Published:** 2025-06-23

**Authors:** Isabelle Moreira, Elizabeth Chodzaza, Mahbub Elahi Chowdhury, Thierno Dieng, Rasheda Khan, Sylvain Landry Birane Faye, Kaosar Afsana, Martha Kamanga, Chifundo Colleta Zimba, Emas Potolani, Kate Ramsey, Joseph Savage, Lynn Freedman, Caitlin Margaret Warthin, Samantha Lobis

**Affiliations:** 1 Averting Maternal Death and Disability Program (AMDD), Heilbrunn Department of Population and Family Health, Mailman School of Public Health, Columbia University, New York, New York, United States of America; 2 School of Maternal, Neonatal and Reproductive Health, Kamuzu University of Health Sciences, Blantyre, Malawi; 3 Health Systems and Population Studies Division, icddr, b, Dhaka, Bangladesh; 4 Centre Régional de Formation de Recherche et de Plaidoyer en Santé de la Reproduction (CEFOREP), Dakar, Sénégal; 5 Laboratoire de Sociologie, d’Anthropologie et de Psychologie (LASAP-ETHOS) FLSH, Université Cheikh Anta Diop, Dakar, Sénégal; 6 BRAC James P Grant School of Public Health, BRAC University, Dhaka, Bangladesh; 7 Midwifery Department, School of Maternal, Neonatal & Reproductive Health, Kamuzu University of Health Sciences, Lilongwe, Malawi; 8 Scope Impact, New York, New York, United States of America; 9 Scope Impact, Helsinki, Finland; Jhpiego, UNITED STATES OF AMERICA

## Abstract

The Emergency Obstetric and Newborn Care (EmONC) Framework has been instrumental in helping countries plan and monitor maternal health services for over 20 years. Given evolving health systems, the changing expectations of both patients and health providers, and the expanded evidence base, the “Re-Visioning EmONC” project was initiated to update this framework to better meet countries’ needs. To understand the needs of its primary intended users, the project used human-centered design (HCD) to conduct in-depth studies in three countries with extensive experience using the EmONC Framework: Bangladesh, Malawi, and Senegal. The study employed HCD methods to conduct interviews, focus groups, and consultative workshops with 337 participants (e.g., health managers, health providers, and service users) across the three countries. Each country team developed their own themes to explore within the boundaries of the overall Re-Visioning EmONC project’s global objectives and developed unique generative activities based on the primary research questions and the category of participants. Multi-stage data analysis was conducted using affinity diagrams and either atlas.ti or Nvivo. Seven key cross-country insights emerged that together can be summarized as follows: when health systems fail, the burden of accessing and providing EmONC shifts to individuals – women, families, and health providers – who must improvise solutions, leading not only to poor quality of care, but also to huge out of pocket expenses, poor wellbeing and a profound mistrust in each other and in the system. The insights informed revisions to the EmONC Framework, including enhanced guidance on context-specific planning, new indicators for facility readiness, incorporation of workforce wellbeing, and increased focus on integrated maternal-newborn care. The HCD approach enabled meaningful integration of user perspectives into the revised EmONC Framework. The revised framework provides a roadmap for strengthening health systems and improving outcomes for women with obstetric complications and small and sick newborns.

## Introduction

In the past two decades, maternal and neonatal mortality have declined in the vast majority of countries around the world. Despite these overall declines, many countries’ progress has stagnated or slowed in recent years [1–3]. The COVID-19 pandemic, economic instability, armed conflict, and climate change have contributed to stalled progress, so now more than ever, countries need tools to help efficiently and effectively address complex development challenges and plan context-specific responses to maternal and neonatal mortality.

The Emergency Obstetric and Newborn Care (EmONC) Framework was originally described in the 1997 “Guidelines for monitoring the availability and use of obstetric services” and updated in 2009 in “Monitoring Emergency Obstetric Care: A Handbook” [4,5]. Over the last 20 years, the EmONC Framework—a set of obstetric and neonatal signal functions organized into levels of EmONC, and a set of indicators—has been used extensively in over 50 countries worldwide to help countries plan, monitor and evaluate EmONC services [6]. The Framework has provided countries with critical information to help develop data-driven approaches to reducing maternal mortality, set targets, and track progress in strengthening EmONC service delivery. At the same time, over the last two decades, country health systems have evolved, the maternal and newborn health (MNH) evidence base has expanded, and a wealth of implementation lessons have been collected about what works and what remains challenging across different settings [7–9].

With these developments, a 5-year project was initiated in 2020 to update the EmONC Framework to better meet countries’ evolving needs. The Re-Visioning EmONC project was led by a global steering committee comprising MNH experts who followed a systematic process to review, rethink, and revise the EmONC Framework. The process employed a human-centered design (HCD) approach to ensure that the re-visioned Framework met the needs of its primary intended users, namely national and subnational government planners and managers responsible for MNH, and the partners that support them. The full process included systematic and scoping literature reviews, a Delphi study, and secondary data analysis [10,11]. In addition, the perspectives of people who had extensive experience using the Framework for planning, monitoring, managing, and providing EmONC services were understood as critical to identifying gaps, challenges and new ideas. EmONC service users’ (women and their partners’) views were also deemed important to take into account. To gain these perspectives, the Re-Visioning project applied HCD to conduct in-depth studies in three countries with extensive experience using the EmONC Framework for planning, monitoring and evaluating efforts to reduce maternal and neonatal mortality: Bangladesh, Malawi, and Senegal (Table 1).

The objectives of the three studies were: to learn from country-level planners and managers, health providers and communities (women and husbands) about their challenges in delivering and accessing good-quality EmONC services; to determine what new areas should be prioritized for inclusion in the revised EmONC Framework; and to gather feedback on how to make the EmONC Framework more useful for planning, implementing and monitoring good-quality, equitable EmONC services. The Re-Visioning EmONC project then used the insights from these studies to develop key concepts or constructs, which were subsequently incorporated into the revised EmONC Framework.

**Table 1:** Use of the 1997 and 2009 versions of the EmONC framework in Bangladesh, Malawi and Senegal.

	Use of the EmONC Framework
Bangladesh	• Conducted EmONC assessments in 1994, 2001, 2006, 2012 [12–20] • In the late 1990s, used the EmONC Framework to inform the redesign of the health system (e.g., planned, established, upgraded, equipped and staffed facilities to ensure geographic coverage of EmONC services; planned training and quality improvement interventions) [21] • Incorporated EmONC indicator data elements into the national health information system • Currently use select EmONC indicators to report on maternal health services in health bulletins used by program managers to monitor service utilization [22,23]
Malawi	• Conducted EmONC assessments in 2005, 2010, 2015, 2021 [24–27] • Integrated EmONC indicators into HMIS • Ministry of Health officials use the EmONC Framework to inform the development of plans, policies and protocols and to monitor the performance of EmONC services • Findings of the EmONC assessments have been used to design maternal and newborn health programs and quality improvement interventions as well as to mobilize resources • Facilities use the EmONC indicators to plan and allocate resources
Senegal	• Conducted EmONC assessments in 2000, 2012, 2016 [28–30] • Used findings of EmONC needs assessments to develop policies, protocols, plans and programs for maternal mortality reduction, and to monitor the performance of EmONC services • Used data from previous EmONC needs assessments to plan and monitor the implementation of a national network of EmONC facilities • Integrated EmONC signal functions and indicators into DHIS2 and currently use the system for routine monitoring of the national EmONC Network • Integrated monitoring of EmONC indicators into basic pre-service and on-the-job training for midwives, doctors and obstetricians/gynecologists

## Methods

The research was led by icddr,b and BRAC University in Bangladesh, Kamuzu University of Health Sciences in Malawi and Centre Régional de Formation de Recherche et de Plaidoyer en Santé de la Reproduction (CEFOREP) in Senegal, with training in HCD and design support from Scope Impact and technical support from Columbia University’s Averting Maternal Death and Disability Program (AMDD). Reporting follows the recommendations for health research involving design put forth by Bazzano et al. [31].

### Study design

The study employed HCD methods in the three countries. HCD is a systematic process for participatory co-creation based on users’ experience to improve existing interventions or innovate novel solutions. The HCD approach was chosen for its strengths in eliciting information that can sometimes be hard to capture using direct questions, sparking new ideas and opportunities, and stimulating participatory engagement in feedback on proposed solutions [32,33]. Qualitative methods including interviews, focus groups and consultative workshops were employed with the primary users of the EmONC Framework (i.e., national and subnational planners and managers who are involved in the planning and monitoring of services), as well as stakeholders who either use or provide EmONC services. Generative design techniques were used to help stimulate discussion and engage participants. These methods include tangible materials, activities and games and are designed to explore user experience at a deeper level, helping to spark open dialogue, foster new ideas, and facilitate conversations [34]. While the tangible materials produced are part of research findings, the critical data are the conversations around their production, such as decisions and explanations that provide new insights.

The studies were conducted in Bangladesh, Malawi and Senegal at varying stages of the overall Re-Visioning EmONC project’s global process: Bangladesh collected their data 01/10/2022–31/03/2023, Malawi collected their data 15/02/2023–30/06/2023, and Senegal collected their data 01/05/2022–01/11/2022. In 2023, individual health providers in Senegal gave input on the feasibility of early indicator concepts in terms of relevance and data collection burden.

Each country team developed their own themes to explore within the boundaries of the overall Re-Visioning EmONC project’s global objectives. Table 2 shows the themes that were explored in each country. Research questions were developed based on these themes.

**Table 2:** Research focus by country.

Country	Specific themes explored
**Bangladesh**	• Experiences and challenges of maintaining 24/7 functioning of EmONC services • How the private sector (nonprofit and for-profit) could be effectively integrated into national plans and strategies for delivering good-quality, equitable EmONC services
**Malawi**	• Challenges of achieving well-functioning Basic EmONC^a^-level facilities • Problems of readiness to perform EmONC • Issues around referral between Basic EmONC and Comprehensive EmONC^b^ facilities
**Senegal**	From the perspective of health providers and government planners: • Description of how the current EmONC system works • Visions of an ideal EmONC system • Needed/wanted information to improve and ensure good-quality EmONC services.
	From the perspective of women and their husbands: • Experiences accessing maternity care • Desired elements of good-quality maternity services

^a^ Basic EmONC facilities should perform the following set of signal functions (clinical interventions): administer parenteral antibiotics, administer uterotonic drugs (i.e., parenteral oxytocin), administer parenteral anticonvulsants for pre-eclampsia and eclampsia (i.e., magnesium sulfate), manually remove the placenta, remove retained products (e.g., manual vacuum extraction), perform assisted vaginal delivery (e.g., vacuum extraction), and perform basic neonatal resuscitation (e.g., with bag and mask) [5].

^b^ Comprehensive EmONC facilities should perform all of the Basic EmONC signal functions (see above) plus the following signal functions: perform surgery (e.g., cesarean section) and perform blood transfusion [5].

In each country, some participants also engaged in co-design by reviewing potential concepts for measurement, refining and providing feedback on proposed indicators (e.g., perceptions of ease/burden of data collection, their utility), and prioritizing which indicators to include in the final set. Participants also provided some feedback on signal functions and levels of care. This article, however, focuses on the insights derived from exploration of the topics outlined in Table 2.

### Study population and sampling

Study participants were purposively selected across the three countries based on the identified themes and their role in the use, provision, monitoring or planning of EmONC services. These included the primary users of the Framework - national policymakers, national and subnational managers – as well as other stakeholders, including health facility managers, health providers (e.g., medical doctors, nurse-midwives, nurses), community representatives, and women who recently gave birth and their male partners.

The study involved 337 participants across the three countries, with 106 participants in Senegal, 157 participants in Bangladesh and 74 participants in Malawi. Overall, 48 participants were interviewed and 289 people participated in 41 workshops/focus group discussions (FGD), as shown in Table 3.

**Table 3:** Number of participants: key informant interviews and focus group discussions/workshops.

Category of participant	Number of participants					
	Bangladesh		Malawi		Senegal	
	KIIs^a^	FGDs^b^ (n = 14)	KIIs	FGDs (n = 11)	KIIs	FGDs (n = 16)
National managers/policymakers	5	21	1	5	2	
Subnational managers	7 (public & private)		3	12		17
Facility managers		18 (public & private)				
Health providers^c^	11	80	5	36	4	20
Transport officers/ambulance drivers				11		
Community representatives	4	11				
Women service users and male partners						58
UN agencies and other government partners			1		3	
Other experts					2	
**Total**	**27**	**130**	**10**	**64**	**11**	**95**

^a^ Key informant interviews = KIIs.

^b^ Focus group discussions = FGDs.

^c^ Health providers included: medical doctors/physicians, nurse-midwives, nurses, clinical officers, obstetrician-gynecologists, pediatricians, neonatologists, other expert clinicians.

In Senegal and Malawi, participants were identified from different subnational areas and brought together for workshops, while in Bangladesh, the workshops were held in the locations where participants were based. In Senegal, a subset of 20 participants were invited back for two additional workshops to review and provide feedback on early versions of the EmONC Framework.

In addition, the study in Bangladesh included observations in four hospitals.

### Data collection and management

For each country, interview and workshop guides were developed by the country research team in collaboration with the Scope and AMDD teams. Unique generative activities (Table 4) were developed for each country based on the primary research questions and the category of participant. To gather participants’ feedback on the draft EmONC Framework, potential EmONC indicators and promising concepts, some techniques were adapted and additional generative techniques were also used (Table 4).

**Table 4:** Generative activities.

Activity	Description	Countries where used	Frequency of use (number of sessions)
**Collage systems map**	Systems mapping enabled participants to show the flow of a system or process. Participants used pre-selected images of places, things, and roles in the health system, thereby creating a collage when mapping the system.	Malawi Senegal	4 FGDs 16 FGDs
**Journey maps**	Journey maps helped to lay out experiences along a time axis and were used to capture health workers’ journeys in providing specific EmONC interventions. Journey maps were also used to explore participants’ thoughts about early concepts being considered for the Framework; this activity enabled participants to walk through an issue and identify important moments for service delivery and data capture that relate to proposed changes to the EmONC Framework.	Malawi Senegal	3 FGDs 2 FGDs
**24-hour clock**	A clock diagram enabled participants to map what occurs during 24-hour cycles of providing care, including highlighting moments when the challenges are the greatest and why.	Bangladesh	14 FGDs
**Persona creation**	Participants created fictional profiles of a typical person who is similar to them, enabling them to speak in the third person about their experiences.	Malawi	3 FGDs
**Organizational typologies**	Organizational typologies were created as fictional health facilities representing different types of organizations in which participants described how they function, enabling the ability to speak more generally about problems and challenges.	Bangladesh	2 FGDs
**Ranking**	Participants were asked to prioritize or rank items that emerged from other generative activities, such as the collage systems map. Participants also used this activity to prioritize components of indicator prototypes or sets of indicator prototypes based on their relative importance using a variety of techniques.	Bangladesh Malawi Senegal	5 KIIs 11 FGDs 16 FGDs
Walk through*	The facilitator talked participants through a framework or an indicator and asked them to provide feedback and reactions throughout the description.	Malawi Senegal	11 FGDs 16 FGDs
Sliders*	Participants evaluated proposed indicators according to certain criteria, such as measurability, actionability, understandability and feasibility. For each criterion, they placed their assessment on a slider that went from negative to positive.	Bangladesh Malawi Senegal	12 KIIs 11 FGDs 8 FGDs
Storyboard*	Participants were asked to review the potential of a new indicator by developing a visual story of how it would work in practice and be used. Each stage from data capture to final use included a simple drawing with corresponding text.	Malawi Senegal	11 FGDs 8 FGDs

* These HCD methods were used for co-creation and prototyping but specific findings are not reported here.

Guides and materials for generative activities were initially developed in English and then translated to relevant languages by the research teams, including French, Wolof, Chichewa and Bangla. The interviews and workshops/FGDs were conducted by members of the research team in each country, with support from trained research assistants in Bangladesh and Senegal. Audio recordings were transcribed verbatim, either by a research assistant or using Sonix.ai transcription and translation. Transcriptions were reviewed for accuracy. Photographs were used to capture the materials produced through generative techniques.

### Data analysis

Country-level analyses first were conducted by each country using affinity diagramming to identify preliminary themes. An affinity diagram organizes ideas, problems, and solutions into related groups with the goal of categorizing and organizing a large body of information into a limited number of logical cohesive groups [34]. A digital whiteboard, Miro, was used to map the information into clusters and name them as themes. Codebooks were then developed from these themes and transcripts were line coded using either atlas.ti or Nvivo, adjusting the codebook as needed based on the transcripts. The teams also reviewed the photographs that captured the tangible artifacts created in workshop sessions, such as collages and journey maps. The digital whiteboard was then used again to map out the emerging themes from each code and to show relationships between the themes. From this analysis, insights were identified. Insights are deeper learnings that emerge from multiple data points, and may help explain latent motives, thoughts, or feelings. Insights are typically not something that research participants readily express in interviews, but rather are identified through continued analysis of findings. As compared to research findings, insights focus on inspiring or driving change, rather than descriptions or explanations about an area of enquiry. Actionable insights for this study focused on findings that could help envision possible opportunities for revisioning the EmONC Framework.

The insights and corresponding text from each country were then used to identify common, cross-country insights and experiences. The text for each was read and concepts and quotes from the text were organized through affinity mapping using Miro, inductively creating new clusters or themes from across the three studies to identify universal learning. Based on these clusters and relationships, overarching insights were developed. The data were then mapped in a framework for each insight by country. As data were mapped and narratives written about the insights based on the data, some insights were combined resulting in a final seven.

### Reflexivity

The initial insights that emerged from this research were developed in each country by the study teams with some support from Scope Impact and AMDD. Each team brought different backgrounds and perspectives to the research.

In Bangladesh, the core research team included four maternal health experts: three with epidemiology and medical backgrounds and one anthropologist. All brought extensive research, program, and advocacy experience in maternal and neonatal health systems and the overall MNH context of Bangladesh. Issues were explored through multiple methods which helped triangulate and validate the findings during the analysis.

The Malawi research team comprised three nurse-midwife researchers trained in public health and an expert in HCD. The midwives brought substantial practical experience from previous work in several maternity units in Malawi. Moreover, two researchers were actively teaching midwifery, including topics specifically related to EmONC. Their familiarity with some participants posed risks of bias which was mitigated through continuous reflection on the impact of their positions on the research.

The complementary profiles of the Senegal research team included an obstetrician/gynecologist with expertise in public health, two medical anthropologists, and a statistician specializing in clinical research, and monitoring and evaluation. The diversity of professional profiles and socio-cultural sensitivity of the team made it possible to engage with a diverse range of participants.

The global analysis was conducted by a four-person team with expertise in public health, obstetrics, social science and HCD. The team brought a wealth of experience accumulated over 25 years of collaborations across numerous countries which enriched perspectives and methodologies, ensuring that the approach was grounded in both rigorous methodologies and real-world perspectives.

### Ethics statement

Written informed consent was obtained from all individuals involved in these studies. All consenting participants were informed that they could refuse to answer any question and were free to stop their involvement in any of the HCD and other research activities at any time. All data are stored securely in accordance with IRB ethical standards and institutional data protection protocols. All transcripts are fully anonymized. Digital files were encrypted and password-protected, with access limited to authorized research team members. Ethical approval was provided by the institutional review boards of Columbia University, icddr,b in Bangladesh, the University of Malawi College of Medicine Research and Ethics Committee (COMREC), and the National Ethics Committee of the Ministry of Health in Senegal (CNERS).

### Inclusivity in global research

Additional information regarding the ethical, cultural, and scientific considerations specific to inclusivity in global research is included in the Supporting Information (S1 Checklist).

## Results

### Cross-country insights

Seven main insights emerged from the three country studies. Their implications for the EmONC Framework are described in the discussion section.

**1) Women's and newborns’ lives are in danger due to health system failures caused by inadequate resources, insufficient planning and management, and a lack of accountability**

The first insight underpins the six other insights. It refers to broader health systems functionality related to planning, management, and financing that participants encounter while implementing EmONC programs or providing services. In all three studies, participants spoke repeatedly about how the lack of systematic, comprehensive planning and management for EmONC within the larger health system puts women's and newborns’ lives at risk.

In all three countries, the haphazard and/or inefficient purchasing and distribution of equipment and medical supplies resulted in under-equipped facilities. In Bangladesh, the problem of ordering and purchasing medical supplies based on the number of beds at facilities and not on the number of people using those facilities can result in over-purchasing of commodities in facilities that have low utilization and under-purchasing commodities in facilities that have high patient volumes that exceed official bed capacity. In Malawi, participants talked about how little funding is allocated to districts for healthcare, and sometimes facilities only receive 10% of their annual operating budget thus crippling their ability to provide effective and good-quality services.

*“There is need for more funding to enable health facilities to have necessary resources and medical equipment such as fuel, vacuum extraction machines, refrigerators to keep oxytocin, batteries for blood pressure machines etc. necessary for their operation.” (Malawi, District Health Management Team)*

Further, even when equipment is available at health facilities, the deployment of human resources is sometimes not aligned. Participants in Senegal spoke about the poor distribution and planning of medical equipment and human resources across health facilities. This can mean that some health facilities designated to provide EmONC have the equipment required to provide EmONC but do not have adequate staffing to use the equipment and other facilities cannot function due to a lack of equipment while the required staff are available.

In government/public health facilities in Bangladesh, specialist medical doctors work only in the mornings and leave in the afternoons and evenings to practice at for-profit private facilities. As a result, government/public facilities are left without medical doctors who can provide EmONC during critical hours, hindering 24/7 access to these services.

The following six insights elaborate on the consequences of these shortcomings and how they affect women, newborns, health providers, and other staff.

**2) Health providers must find quick, often imperfect, solutions in chaotic, under-resourced health facility environments, sometimes compromising the quality of care**

When health facilities do not have sufficient staffing or the right equipment, drugs, and supplies, health providers are forced to provide EmONC under inadequate conditions. This is not only stressful for health providers but impacts the quality of care they can provide to women and newborns with complications. In all three countries, there were numerous examples of health workers facing these kinds of challenges.

In Senegal, midwives described how they are often alone in Basic EmONC facilities and must manage all of the clinical and administrative tasks on their own. They explained that at a delivery, they must care for both the mother and newborn at the same time while being responsible for delivering other services at the facility. Health providers in Senegal also spoke of working in facilities meant to provide Comprehensive EmONC, but that lack blood, oxygen and well-functioning resuscitation equipment, which compromises the quality of care they can provide.

In Bangladesh, after 2 pm and particularly during the night, holidays, and weekends, specialized obstetricians and anesthesiologists may not be present onsite at district and subdistrict public facilities designated to provide EmONC. In addition, the on-call system for coverage of EmONC services when specialized cadres are not onsite is not functional. In such critical moments, nurses are expected to provide care for patients in consultation with duty doctors or they are compelled to refer patients to tertiary-level facilities, nearby district hospitals, or private facilities for definitive treatment. Another challenge is that essential emergency medications are often not available 24/7. A nurse working at a facility that is supposed to provide Comprehensive EmONC shared that:

*“Night shift is dangerous...the pharmacies (at the facility) remain closed. In our area, the pharmacies (outside) remain closed as well. Therefore, how would they manage the medicines prescribed by us? So, as we have shortages of supplies and as the pharmacies are closed during the night, this is a huge problem. If the delivery takes place at night, and if we need oxytocin, we are in serious trouble...” (Bangladesh, Nurse)*

In Malawi, nurse-midwives described how difficult it is to provide care to so many women at once. They spoke about insufficient time to provide good-quality care according to professional standards. They also explained how handling so many patients simultaneously can negatively affect their ability to make competent decisions. A nurse-midwife explained:

*“We do shortcuts when managing women with complications because of increased workload which affects the quality of care.” (Malawi, Nurse-Midwife)*

**3) Health providers are sometimes worried, afraid, and lack confidence to perform certain EmONC clinical interventions because there are no adequate support systems to maintain their skills and ensure competence**

Health providers described situations when they do not feel comfortable or confident providing certain EmONC services. They felt that it was assumed that they should be providing these services, yet they are not given opportunities to practice or improve their skills, do not receive regular mentorship and support, and/or do not have access to current clinical protocols and guidelines.

Providers in Bangladesh and Senegal spoke about insufficient practical skills among new graduates (intern doctors in the case of Bangladesh), while providers in Malawi asked for more in-service training/practice. In Senegal, insufficient Basic EmONC training was raised, particularly for midwives who attend the many new unregulated and poor-quality midwifery schools that have recently opened across the country. In addition, many midwives and doctors talked about being afraid to treat small and sick newborns due to insufficient practical training and because of the ‘social gaze’ of families and professional colleagues, mostly criticizing their competencies. In Bangladesh, newly appointed medical doctors (with urban backgrounds) who are placed in rural facilities without sufficient work and practical experience in that context, do not want to take any risks when managing women with obstetric complications. To avoid risk, they rely on referring women to other health facilities.

In Malawi, nurse-midwives felt that they need more skills drills at the facility level to practice, especially for managing cases of eclampsia. This especially concerns them when they receive a new brand or type of drug without sufficient guidance and training because if they administer the drug incorrectly and there is an adverse event, they fear sanctions from regulatory bodies.

In Bangladesh and Malawi, providers without official authority or training might have to perform EmONC (i.e., assisted vaginal delivery, manual removal of placenta) without adequate support due to shortages of more senior and/or experienced and well-trained providers in facilities. Nurses in Bangladesh described how they must handle obstetric emergencies, especially at night, with limited or no support from trained doctors. In Malawi, due to severe staffing shortages, sometimes providers who are not trained in EmONC must step in to provide care during emergencies. When they are unable to provide definitive care, they must refer patients to other health facilities despite knowing that the referral system is unreliable.

*“..better to leave these things to senior members or just refer the case, although it is an emergency.” (Malawi, Nurse-midwife technician)*

**4) Continuous care for women and newborns at facilities and during referral is disrupted due to poor system readiness, which poses mental and physical danger to women, babies, health providers and ambulance drivers**

When facilities and health providers are unprepared to provide EmONC 24/7, many women and newborns with complications must be referred to other facilities to receive care. Interfacility emergency transfer of women and newborns with complications must be organized in a way that ensures continued care during the journey and minimizes delays. However, participants in the three countries shared that all aspects of the journey between facilities put all of the people involved at risk of physical harm and mental anguish. This includes women and newborns with complications, their families as well as ambulance drivers.

A shortage of EmONC-related specialists in public facilities in Bangladesh, especially on nights, weekends, and holidays, causes many women and newborns with complications to be referred. Due to the lack of a cohesive, planned emergency referral system, women and newborns are often referred to other facilities that are also dysfunctional, and they are transported in ambulances that are not well-equipped to provide emergency care onboard.

*“Those...with severe complications...we refer....no cesarean sections take place at all during the weekends and holidays. The Gynecologist and the Anesthetist remain on leave during these days. Therefore, if any such women come during these days, we just refer them...” (Bangladesh, Nurse)*

Nurse-midwives in Malawi spoke about sometimes not feeling confident to treat women with severe pre-eclampsia and eclampsia. Instead, they pass off those cases to senior health providers or refer those women to other facilities, even though they realize it is an emergency. Then, because of staffing shortages, nurse-midwives explained that usually there are no health providers available to accompany women and newborns in the ambulance, leaving the driver alone with patients. Ambulance drivers explained that they are not medically trained but are sometimes required to provide care to women and babies at the same time as driving the ambulance in challenging terrains. In Malawi, there are many factors that contribute to a dysfunctional referral system including: poorly serviced and ill-equipped ambulances frequently running on low fuel tanks and protracted procurement procedures for ambulance repair and maintenance. Additionally, even when the referral system is functional, geographic obstacles such as poor road conditions and rivers during the rainy season prevent ambulances from quickly transporting women and newborns during emergencies.

In Senegal, participants spoke about traveling far to receive care at facilities that they discover are unprepared to provide EmONC. They then need to travel even farther to another facility to receive care, causing significant delays in receiving emergency care which puts their lives at risk. According to service users living in rural areas in southeast Senegal, some families travel more than 70 km to get to a health center and then if the newborn has complications, there is no resuscitation equipment and no qualified staff to provide care. It is then necessary to travel even farther towards the city to find a functional EmONC facility.

**5) Systems fail newborns due to lack of focus on the dyad and limited integration of maternal and newborn services**

In Bangladesh and Senegal, participants spoke about logistical, managerial, and clinical challenges that inhibit patient-centered, integrated care for women and their newborns, thus leading to poor quality of care.

Sometimes facilities are not set up in a way that promotes interactions between women and their newborns. In Bangladesh, participants spoke about how some maternity and newborn units are on different floors and in some cases, are located in different buildings.

“*...how difficult is it for a cesarean section mother to go to the fourth floor (from first floor) to breastfeed the baby – just tell me!... There is no elevator! It has been out of order for a long time” (Bangladesh, Nurse)*

In Senegal, providers believe that the government’s past focus on women with obstetric complications has led to less attention on small and sick newborns. As a result, skills are limited, essential equipment is often unavailable or nonfunctional, commodities are scarce, and newborn corners are often inoperable.

*“After delivery, the newborn is somewhat forgotten in the delivery room. It seems like EmONC just stops when the woman gives birth.” (Senegal, Pediatrician)*

Similarly in Bangladesh, participants highlighted outdated and poorly functioning equipment in the special newborn care units and a shortage of newborn specialists.

**6) Health providers’ wellbeing suffers as a result of poor support from the system and strained relationships with communities**

Throughout the sessions, health providers across the three countries described their stress, and poor mental health and wellbeing due to providing care in challenging health system conditions. Difficult relationships with communities sometimes worsen this stress.

Providers bemoaned insufficient staffing levels which create a tiring and sometimes overwhelming workload which is further exacerbated when they must work without essential equipment and supplies. Malawian nurse-midwives described their frustration with having to provide care without proper training and adequate staffing, and how that leads to dissatisfaction with their jobs. Nurses in Bangladesh felt overwhelmed and frustrated by having to handle emergencies on their own without clinical support from medical doctors, particularly at night. In Senegal, providers described feeling tired from too many tasks and having no time to rest while trying to do the maximum to save maternal and newborn lives. They described feeling further demoralized when maternal or newborn deaths occur despite their efforts. They also do not feel supported by their managers when they face criticism from the community following adverse events. Poor relationships with the community can further impact providers’ well-being. For example, Bangladeshi health providers fear physical attacks from the community if something goes wrong when they are managing a complication.

Yet, providers felt that their pleas for support are largely unheard. In Bangladesh, nurses reported feeling ignored by their supervisors when making complaints about work-related problems or sharing potential solutions that could reduce their workload and alleviate their stress. Senegalese health providers, especially those working in rural areas, desire support from their managers for their well-being.

*“…I know I have the skills to do the work expected of me, but I need psychological support” (Senegal, words of a doctor reported by his regional chief physician).*

**7) During emergencies, women and families struggle to mobilize the necessary funds and resources for medication, blood transfusion, and referral transport**

Across the three countries, health providers and service users alike described how those seeking EmONC services often have to take on unexpected roles, creating serious delays in the receipt of care. This includes mobilizing funds for large out-of-pocket expenses and then seeking out and procuring critical items or services such as emergency drugs, vehicles for transport, as well as finding blood donations and transfusion supplies. Families experiencing emergencies sometimes go to extreme lengths to pay for aspects of care that should be part of entitlements to free EmONC services.

In Bangladesh and Senegal, providers and family members spoke of the high costs encountered when medicines and essential commodities are out of stock in public facilities. In these situations, they have to find and pay for essential supplies at private pharmacies where costs are higher. Service users in Senegal described how sometimes they have to make difficult decisions about which medicines to buy given their limited funds.

Across the three countries, people spoke of the costs for referral transport when ambulances are unavailable or there is no fuel. Providers in Malawi described how ambulances are rarely on site or nearby and often lack fuel, resulting in families having to find and pay for expensive taxis despite expectations of free referral. In Senegal and Bangladesh, emergency referral transportation is not free, leaving families to face additional costs when a referral is required. Women service users and their partners in Senegal spoke about having to buy fuel for ambulances or paying for private transportation, such as taxis, when ambulances are unavailable.

*“I recently saw a man whose wife was the victim of transportation problems. His wife died because of that, because he had a money problem of 10,000 francs to pay the driver. So he went to get the money, and came back too late. So eventually he took a long time and his wife died. I saw him crying. So these are lessons to take into account.” (Senegal, Husband of EmONC service user in rural area)*

In Bangladesh, people who are poor are often unable to afford the cost of government ambulances and thus struggle to complete referrals.

Furthermore, in Bangladesh, providers and managers described how family members bear the primary responsibility for arranging blood transfusions due to staffing shortages, especially at night. This forces family members to sometimes seek out private services and find blood donors themselves.

When families must mobilize money to pay for commodities that are essential to the delivery of EmONC, not only are those critical services delayed, some poor families may be pushed further into poverty. Providers in Bangladesh described the impoverishing costs families can incur in emergencies, explaining that many rural families might spend all the money they have to travel to facilities without anticipating additional costs. Often, when families expect to receive poor quality care at public facilities that rarely function 24/7, families may choose to spend their last resources on seeking health services at private facilities instead.

*“....You may say people are not poor, but people are poor. It often happens that they sell their last piece of land to have the cesarean section at the private facilities The family then literally manages the money by great struggle...” (Bangladesh, Senior Family Welfare Visitor)*

### Key country-specific insights

In addition to the cross-country insights, each country team had specific insights that were found to be critical and/or unique to their context. These specific insights also informed the Re-Visioning EmONC project. They include:

- The unavailability of 24/7 EmONC services in public facilities at the sub-district and district levels pushes women to the private sector (Bangladesh)
- A complex array of factors drive the overuse of cesarean sections, especially in private (for-profit) facilities (Bangladesh)
- Private (for-profit) facilities often fail to report data to the HMIS due to fear of losing their license and potential tax liabilities (Bangladesh)
- Communication gaps and interpersonal misunderstandings among health providers across different levels of facilities can erode relationships and create additional delays in the emergency referral system (Malawi)
- Women service users and their male partners lament that health providers do not communicate or show empathy towards them which leads to conflict and the desire to not use the facility in the future (Senegal)

Detailed reports have been published and disseminated locally in each country [35–37].

## Discussion

In public health and the health sector, there has been increasing application of HCD for innovation and improvement of health service delivery, health facility design, education, counseling, healthcare products, and digital applications, among others, to meet the needs of both health service users and health providers [32,38]. HCD has been used in the design of clinical decision support systems or data capture at the patient level, such as electronic medical records or patient surveys [38–41]. In one example, HCD was used to understand the challenges with using data for decision-making in expanded immunization programs. However, based on a review of the literature, HCD has not been previously applied to the design of monitoring frameworks and indicators for health system planning and management. Applying the HCD approach enabled the Re-Visioning EmONC project to understand and prioritize the needs, experiences, and preferences of national and subnational planners and managers, health providers, and EmONC service users. Involving these groups in the revisioning process aimed to make the EmONC Framework more relevant and effective, addressing real-world challenges and ensuring greater usability. This approach also fostered empathy and understanding, leading to recognition and acknowledgment of the pressures and challenges faced by those managing, providing and using EmONC services in constrained health systems.

The teams in Bangladesh, Malawi and Senegal received invaluable input from more than 300 participants who described their challenges in planning and managing EmONC services, as well as delivering and/or accessing good-quality EmONC services. The seven cross-country insights as well as the unique country-specific insights drawn from these studies together can be summarized as follows: **when health systems fail, the burden of accessing and providing EmONC shifts to individuals – women, families, and health providers – who must improvise solutions, leading not only to poor quality of care, but also to huge out of pocket expenses, poor wellbeing and a profound mistrust in each other and in the system.**

The insights from these studies were transformed by the Re-Visioning EmONC project into key concepts (to aid in planning) or constructs (for specific indicators). A subset of participants also provided inputs to the concepts and the indicators, although this information is not reported here. Participants’ contributions were incorporated into the revised EmONC Framework and accompanying Guide as follows: [6] (S1 Fig; S2 Fig)

**1. More guidance on planning context-specific good-quality, efficient, equitable EmONC services within the larger health system**: The foundational principle of the revised EmONC Framework is: “Every pregnant woman and every newborn who experiences complications must have access to the right care in the right place at the right time to survive and thrive.” The revised EmONC Framework has therefore been designed as a tool to help health system planners, managers and implementers to systematically assess existing systems, facilities, and needs – and, to use that information to build a locally-driven vision of the future health system and how to make that vision a reality. This includes deciding which facilities should be appropriately staffed, equipped and supported to deliver EmONC in the short-, medium- and long-term, based on the geography of the country (i.e., topography and road infrastructure) and travel time to facilities, population density, capacity of facilities, patterns of inequity, and women’s choices about where they want to give birth. A prerequisite for planning and implementing this vision of EmONC includes having strong information systems that are inclusive of the private sector. The guidance will recommend, as much as possible, that the EmONC Framework be integrated into routine information systems.
**2. Incorporation of indicators measuring facilities’ readiness to provide EmONC with a particular focus on human resources, commodities and emergency referral:** Health facilities’ inability to manage women and newborns with complications because they do not have critical drugs, supplies and equipment and because they are understaffed, means even more reliance on referral systems to get patients to definitive care. Yet the referral systems are often inefficient and poorly functioning. To address these fundamental and frequently mentioned challenges, the revised EmONC Framework will include indicators to monitor: the adequacy of skilled health personnel to deliver EmONC 24/7, the availability of key tracer commodities required for EmONC and the readiness of facilities to support emergency referral (including care during transport) between health facilities.
**3. Incorporation of workforce wellbeing:** Health providers in the three country studies spoke about how stressful, frustrating and demoralizing it is to attempt to provide EmONC when they are short-staffed, unsupported and do not have key commodities and equipment. To ensure that health providers’ wellbeing is included in planning and monitoring the EmONC system, the revised EmONC Framework will include an indicator and additional resources on workforce wellbeing. Incorporating workforce wellbeing ensures the sustainability of EmONC services, addressing burnout, and improving retention among health providers.
**4. Incorporation of a comprehensive measure of women’s experiences during childbirth:** To ensure that planning processes are responsive to women’s needs and address their concerns, the revised EmONC Framework will include the Person-Centered Maternity Care Scale (PCMC) [42,43]. The PCMC Scale measures women’s experiences during childbirth with specific domains on communication and autonomy, dignity and respect, and supportive care.
**5. Increased focus on integrated care for women and newborns:** The 2009 EmONC Framework focused predominantly on emergency care for women, with less emphasis on newborns. The revised EmONC Framework will now incorporate a full set of tracer interventions for the care of small and sick newborns into the EmONC signal functions. This integration marks a paradigm shift from the earlier EmONC Framework, ensuring a more holistic approach to maternal and newborn care. By having an integrated set of signal functions and indicators for obstetric and neonatal care, experts, planners and managers from both specialties (maternal and newborn) will be brought together to collaborate on implementation in new ways. In addition, because of the known benefits of keeping newborns, including those born small and sick, together with their mothers and families, the revised EmONC Framework will include a focus on whether families have 24/7 access to newborns who require inpatient care.

There were two main methodological considerations for these studies and this process. The first is that each of the studies occurred at different points during the broader global Re-Visioning EmONC process. This meant that as conversations progressed and insights emerged from earlier country studies and the global process, these themes could be explored more deeply in the country studies that followed. In addition, we were able to learn which of the HCD-related techniques worked well for certain topics. This type of iterative approach, a fundamental characteristic of HCD, was a benefit to the global Re-Visioning EmONC process. But, it also meant that the overall methodology varied slightly across studies and later country studies benefited from and built on the methodological experiences of earlier studies. Consequently, there may have been more emphasis on what emerged in earlier countries and there may have been important topic areas that were missed. The second methodological consideration is that the scope and types of participants included in each of the studies differed. EmONC systems and services were explored more broadly in Senegal and the focus was narrowed in Malawi and Bangladesh to focus on particular unique aspects of EmONC in those contexts. In addition, only Senegal included service users, while Bangladesh included community representatives, and Malawi included neither. Therefore, our ability to explore and understand the experiences and perspectives of those actually using the services in Malawi and Bangladesh was limited to what health system actors reported observing. Despite these methodological considerations, the experiences and insights that were captured in these three studies likely reflect the reality of many others in low and middle-income settings who are working toward the reduction of maternal and newborn mortality. Overall, these studies were critical in shaping a revised EmONC Framework that is both contextually relevant and reflective of the realities of delivering and utilizing EmONC services globally.

## Conclusion

The findings from these three country studies were critical to the Re-Visioning EmONC process. The use of HCD allowed the voices of future users of the revised EmONC Framework to be meaningfully integrated, ensuring that the Framework is rooted in the lived realities of those delivering and accessing EmONC services. Beyond contributing to the global Re-Visioning EmONC project, the insights gained in Bangladesh, Malawi, and Senegal have already informed national planning processes, highlighting the immediate applicability and relevance of this work.

The iterative and context-specific approach adopted in these studies underscores the importance of tailoring global health frameworks to diverse local contexts. The Re-Visioning EmONC project has not only advanced the conceptualization of a more integrated and inclusive EmONC Framework but also set a precedent for the application of HCD in developing health system planning and monitoring tools. By addressing critical areas such as equitable service delivery, integrated maternal and newborn care, system readiness, workforce wellbeing, and experience of care, the revised Framework provides a roadmap for strengthening health systems and improving outcomes for women and newborns.

Ultimately, the project’s outcomes reflect a commitment to co-creating solutions with stakeholders, fostering greater trust, and driving sustainable improvements in EmONC services. While the methodology and scope varied across the three countries, the collective insights provide valuable lessons for future initiatives, emphasizing the need for inclusive and adaptive approaches in health system strengthening. The revised EmONC Framework is poised to serve as a vital tool for national and subnational policymakers and managers in their ongoing efforts to achieve equitable and high-quality care for all mothers and newborns.

## Supporting information

S1 Checklist PLOS Questionnaire on Inclusivity in Global Research. (DOCX)

S1 Fig Revised emergency obstetric and newborn care (EmONC) signal functions and levels of care. (PDF)

S2 Fig Revised emergency obstetric and newborn care (EmONC) domains and indicators. (PDF)

S1 Data Codebooks and Themes (country-level analyses). (PDF)

S2 Data Affinity Diagrams (cross-country analysis). (PDF)
