# Supplementary material for: Using human-centered design to re-vision the emergency obstetric and newborn care framework: Insights from Bangladesh, Malawi and Senegal
Source: PLOS Glob Public Health. 2025 Jun 23;5(6):e0004771. doi: 10.1371/journal.pgph.0004771 (PMC12185017; doi:10.1371/journal.pgph.0004771)
Supplement: S1 Fig — (PDF) [file pgph.0004771.s002.pdf]

Revised EmONC signal functions, organized into levels of EmONC

	Obstetric 🡻	Neonatal 🡻
**Level 1 - Basic EmONC**	• Administer medications to treat PPH<br>• Administer parenteral antibiotics<br>• Administer magnesium sulfate<br>• Remove retained products of conception<br>• Perform manual removal of placenta<br>• Perform vacuum assisted vaginal birth<br>• Provide IV fluid replacement therapy<br>• Administer first dose of antenatal corticosteroids prior to referral	• Perform newborn resuscitation with bag and mask<br>• Initiate and support early and exclusive breastfeeding<br>• Administer parenteral antibiotics<br>• Practice immediate kangaroo mother care for preterm and LBW infants<br>• Administer oxygen therapy with pulse oximetry for stabilization and transportation
	• Arrange ambulance, with trained and equipped provider, to a facility that can provide definitive care	

	Obstetric 🡻	Neonatal 🡻
**Level 2 - Comprehensive EmONC**	• Administer medications to treat PPH<br>• Administer parenteral antibiotics<br>• Administer magnesium sulfate<br>• Remove retained products of conception<br>• Perform manual removal of placenta<br>• Perform assisted vaginal birth<br>• Provide IV fluid replacement therapy<br>• **Administer antenatal corticosteroids**<br>• **Perform cesarean section**<br>• **Perform blood transfusion**	• Perform newborn resuscitation with bag and mask<br>• Initiate and support early and exclusive breastfeeding<br>• Administer parenteral antibiotics<br>• Practice immediate kangaroo mother care for preterm and LBW infants<br>• **Provide thermal care with radiant warmer or incubator**<br>• **Administer oxygen therapy with pulse oximetry**<br>• **Provide CPAP**<br>• **Provide phototherapy**<br>• **Perform blood transfusion**<br>• **Enable assisted feeding with expressed breast milk with cup and spoon and tube feeding**<br>• **Provide IV fluids**
	• Arrange ambulance, with trained and equipped provider, to a facility that can provide definitive care	

	Obstetric 🡻	Neonatal 🡻
**Level 3 - Intensive EmONC**	• Administer medications to treat PPH<br>• Administer parenteral antibiotics<br>• Administer magnesium sulfate<br>• Remove retained products of conception<br>• Perform manual removal of placenta<br>• Perform assisted vaginal birth<br>• Provide IV fluid replacement therapy<br>• Administer antenatal corticosteroids<br>• Perform cesarean section<br>• Perform blood transfusion<br>• **Provide intensive level organ support**	• Perform newborn resuscitation with bag and mask<br>• Initiate and support early and exclusive breastfeeding<br>• Administer parenteral antibiotics<br>• Practice immediate kangaroo mother care for preterm and LBW infants<br>• Provide thermal care with radiant warmer or incubator<br>• Administer oxygen therapy with pulse oximetry<br>• Provide CPAP<br>• Provide phototherapy<br>• Perform blood transfusion<br>• Enable assisted feeding with expressed breast milk with cup and spoon and tube feeding<br>• Provide IV fluids<br>• **Perform mechanical ventilation**<br>• **Perform screening and treatment for retinopathy of prematurity**

Version June 2025
