# Supplementary material for: Using human-centered design to re-vision the emergency obstetric and newborn care framework: Insights from Bangladesh, Malawi and Senegal
Source: PLOS Glob Public Health. 2025 Jun 23;5(6):e0004771. doi: 10.1371/journal.pgph.0004771 (PMC12185017; doi:10.1371/journal.pgph.0004771)
Supplement: S2 Fig — (PDF) [file pgph.0004771.s003.pdf]

Revised emergency obstetric and newborn care (EmONC) indicators

Domain 1: Structure

Equitably distributed well-functioning EmONC facilities

Domain 2: Process

People's interaction with the system

Domain 3: Outcomes

Effectiveness of care

Domain 1: Structure - **Equitably distributed well-functioning EmONC facilities**

**Availability**	**1a.** Progress toward long-term goal for EmONC availability
	**1b.** Progress toward interim goal for scaling up EmONC availability
**Adequate conditions to provide good quality care ("readiness")**	**2.** Basic infrastructure
	**3.** Equipment, drugs & supplies
	**4.** Health workforce adequate for caseload*
	**5.** Emergency referral readiness*
**Accessibility**	**6.** Home to Comprehensive EmONC within 1hr

Domain 2: Process - **People's interaction with the system**

Domain 3: Outcomes - **Effectiveness of care**

** Provisional; further methodological development and testing required*

Domain 1: Structure - **Equitably distributed well-functioning EmONC facilities**

Domain 2: Process - **People's interaction with the system**

**Place of delivery**	**7.** Institutional delivery rate
**Met need**	**8.** Met need for emergency obstetric care
**Clinical appropriateness of care**	**9.** Cesarean section as a proportion of all expected births
**Experience of care**	**10.** Person-centered maternity care
	**11.** Togetherness - 24/7 family access to inpatient newborn care unit*
**Health workforce wellbeing**	**12.** Health workforce wellbeing*

Domain 3: Outcomes - **Effectiveness of care**

** Provisional; further methodological development and testing required*

Domain 1: Structure - **Equitably distributed well-functioning EmONC facilities**

Domain 2: Process - **People's interaction with the system**

Domain 3: Outcomes - **Effectiveness of care**

**Impact on maternal mortality in health facilities**	**13.** Institutional maternal mortality ratio
	**14.** Direct obstetric case fatality rate
**Impact on stillbirths and newborn mortality in health facilities**	**15.** Intrapartum stillbirth and very early neonatal death rate
	**16.** Neonatal inpatient mortality rate
