# Supplementary material for: Using human-centered design to re-vision the emergency obstetric and newborn care framework: Insights from Bangladesh, Malawi and Senegal
Source: PLOS Glob Public Health. 2025 Jun 23;5(6):e0004771. doi: 10.1371/journal.pgph.0004771 (PMC12185017; doi:10.1371/journal.pgph.0004771)
Supplement: S1 Data — (PDF) [file pgph.0004771.s004.pdf]

Code books and/or themes used for the country-level analyses: Bangladesh, Malawi and Senegal

Bangladesh

All current codes

Ambulance Service_FRUSTRATION

All the information related to Ambulance, the availability of it, the access of it to the patients, the functionality of it, the required need and and frustration related to this issues

This code will apply for all the health facilities - DH, MCWC, UHC, Private facility

---

Blood management_STRESS

- discussion related to family member's involvement in collecting the blood (either they give the blood or collect it from other institution)
- their money spent in collecting the blood
- suggestion related to involving community to get prepared with blood (during ANC counselling or in any other ways) needing during the delivery etc will be coded here

Discussion related to what providers do in terms of helping the patients' families in collecting blood

---

Blood transfusion System_FRUSTRATION

All the information related to blood bank in the facility:

- if the blood bank is available
- if it is functional
- if functional what activities they carry out
- what are they supposed to do but can not and why,
- what are their suggestion and demand
- from where do they collect the blood when needed (the Community's and provider's personal involvement will not be coded here)
- All the suggestions related to how the blood transfusion system can be made workable in the health facility
- Discussion related to providers and managers expressing frustration in terms of blood transfusion system in the facility
- This code will apply for all the health facilities - DH, MCWC, UHC, Private facility

---

C-section_Community demand_CONCERN

Many providers showed their dissappointment talked that there is a huge demand for c-section from the community ingoring the suggestion of the providers. All these sort of information will be coded here

---

C-section_Emergency/necessary/unnecessary

Information related to emergency and necessary and unnecessary c-section, definition of it, understanding of it and suggestion related to how to retain unnecessary c-section will be coded here.

Central and local level management_FRUSTRATION

- Informing higher authority about local problems
- suggestion related to who can push or provide pressure
- problems why higher authority cannot take action
- suggestion how to make this happen
- their involvement in recruitment and transfer which puts health facility in jeopardy
- recruitment process not taking in time, local recruitment process in their hands
- their power play (lobbying, advocacy in recruitment and transferring) which is often very devastating for the health facility

(This section may be sometimes recoded as 'Problems at the central level' and 'Problems with local level management')

- Local lebel's management involvement in in recruitment process, in outsourcing, in taking care of non-functional equipment's

---

Community awareness

- Information related to community's knowledge
- Information related to suggestion how and where to make the community aware
- Women and newborn seeking care to facilities with severe complications after developing the complications at home or at private facilities
- This code will also incorporate information related to when community people bypassing Public facility and going to private facilities becasue they assume the quality of care is better in private facility

---

Local_coordination_Health & management body_FRUSTRATION

Collaboration and coordination among all the public facilities (from cc to MCWC/DH) and all the administrative bodies (Civil Surgeon, DDFP, UNO, DC, Mayor) to make the service available for community

---

Coordination_Public Private

All Kinds of collaboration (referral), co-ordination (Training to Private facility staff) will be coming here

---

Country context

- Discussion related to overall situation in the country in terms of health, maternal health, education, age at marriage, economy and so on
- Any issues related to Covid 19

Demand Side Financing

Ensuring 24/7 EmONC_DISSAPOINTMENT

All the discussion related to ensuring 24/7 EmONC care will be coming here - unavailability of physicians, blood, medicines, tests, referral etc. and suggestion to ensure 24/7 EMONC service

Please note that when this coding will talk about unavailability of any particular physicians, that part will be recoded for that physician's code (obs gyn consultant etc).
Also, if they talk about blood as a barrier, when this will be coded here, that section will also be recoded for blood related code/s which suits best.

Facility readiness_FRUSTRATION

Facility readiness will be understood from so many codes, however, this code will apply for those discussion when informants expressed their frustration about health facility readiness in general

---

Family Welfare Centre

Any discussion related to FWC

Frustration-stress-concern

This code will incorporate all the discussion related to any issues towards which informants literally and verbally mentioned about frustration, stree and dissapointment (HR, Blood bank, Technology, tests, medicines AND Community's negative perceptions and experiences regarding any issues)

--

HR_Anesthetist

Discussion related to Anesthetist - how many are available, how many posts are vacant, how many they think its appropriate to assign for that particular health facility/unit/department, and any other problems and issues related to Anesthetist will be coded here.

This code will apply for all the health facilities - DH, MCWC, UHC, Private facility

---

HR_Community Skilled Birth Attendent

Discussion related to CSBA - how many are available, how many posts are vacant, how many they think its appropriate to assign for that particular health facility/unit/department, and any other problems and issues related to EMO will be coded here.

This code will apply for all the health facilities - DH, MCWC, UHC, Private facility

---

HR_Emergency Medical Officer

Discussion related to EMO (emergency medical officer) - how many are available, how many posts are vacant, how many they think its appropriate to assign for that particular health facility/unit/department, and any other problems and issues related to EMO will be coded here.

This code will apply for all the health facilities - DH, MCWC, UHC, Private facility

---

HR_EOC trained Doctor and Nurse

Discussion related to all the EOC trained doctor and nurse and FWVs - how many are available, if they think this is important that doctors and nurses and FWVs gets this training and that more doctors and nurse and FWVs with EOC training should be in their facility, how they value the training and so on will be coded here.

Also any discussion related to EOC training will be coded here

This code will apply for all the health facilities - DH, MCWC, UHC, Private facility

---

HR_Family Welfare Visitor

HR_Indoor Medical Officer

Discussion related to IMO (Indoor Medical officer) - how many are available, how many posts are vacant, how many they think its appropriate to assign for that particular health facility/unit/department, and any other problems and issues related to IMO will be coded here.

This code will apply for all the health facilities - DH, MCWC, UHC, Private facility

---

HR_Medical Officer

Discussion related to MO (Medical Officer) - how many are available, how many posts are vacant, how many they think its appropriate to assign for that particular health facility/unit/department, and any other problems and issues related to MO will be coded here.

This code will apply for all the health facilities - DH, MCWC, UHC, Private facility

---

HR_Medical Officer-Clinic

Discussion related to MO-clinic - how many are available, how many posts are vacant, how many they think its appropriate to assign for that particular health facility/unit/department, and any other problems and issues related to MO-clinic will be coded here

---

HR_Neonatologist

Discussion related to Neonatologist - how many are available, how many posts are vacant, how many they think its appropriate to assign for that particular health facility/unit/department, and any other problems and issues related to EMO will be coded here.

This code will apply for all the health facilities - DH, MCWC, UHC, Private facility

---

HR_Nurse and midwives

Discussion related to Nurse and Midwives - how many are available, how many posts are vacant, how many they think its appropriate to assign for that particular health facility/unit/department, and any other problems and issues related to nurse and midwives will be coded here.

This code will apply for all the health facilities - DH, MCWC, UHC, Private facility

---

HR_Obs Gyn Specialists

Discussion related to Obs Gyn Specialists Consultant - how many are available, how many posts are vacant, how many they think its appropriate to assign for that particular health facility/unit/department, and any other problems and issues related to Obs Gyn will be coded here.

This code will apply for all the health facilities - DH, MCWC, UHC, Private facility

---

HR_Other staff

Discussion related to support staff (Ayah, cleaners, technicians at any level, drug dispenser, gardner,) - how many are available, how many posts are vacant, how many they think its appropriate to assign for that particular health facility/unit/department, and any other problems and issues related to other staff will be coded here.

This code will apply for all the health facilities - DH, MCWC, UHC, Private facility

---

HR_Out sourcing

HR_Security Service_FEAR

Discussion related to Security Service - how many are available, how many posts are vacant, how many they think its appropriate to assign for that particular health facility/unit/department, and any other problems and issues related to EMO will be coded here.

This code will apply for all the health facilities - DH, MCWC, UHC, Private facility

---

Indicator 1

At least 5 EmOC facilities (including at least 1 comprehensive facility) for every 500,000 population

---

Indicator 2

- All subnational areas have at least 5 EmOC facilities (including at least 1 comprehensive facility) for every 500,00 population
- Discussion related to people living in hard to reach areas - why the delay take place, what needs to be done for this community etc.

Indicator 3

Proportion of all births in EmOC facilities

Indicator 4

Met need for EmOC: proportion of women with major direct obstetric complications who are treated in EmOC facilities

---

Indicator 5

The estimated proportion of births by caesarean section in the populations is not less than 5% or more than 15%

Indicator 6

Direct obstetric case fatality rate. The case fatality rate among women with direct obstetric complications in EmOC facilities is less than 1%

Indicator 7

Intrapartum and very early neonatal death rate

- Discussion related to types of still birth - macerated and fresh still birth, keeping the data on this, problems related to not keeping the data, will be coded here
- Discussion related to birth weight of the newborn

Indicator 8

Proportion of maternal deaths due to indirect causes in EmOC facilities

---

Infrastructure

- Public Facility infrastructures - buildings, beds and related issue, toilet and water supply, food, space, space for maintaining privacy for women, rooms for physicians and support staff
- This code will apply for all the health facilities - DH, MCWC, UHC, Private facility
- Please note that the infrastructure buildings for the residential physicians will not be coming here. That will be coded under the code for that particular physicians and for " **Unavailability of Providers"**

Knowledge_ EmONC Framework

This will code the information if the informants know about EmONC framework – the indicators and Signal functions

---

Medicine & drug availability_FRUSTRATION

- Unavailability of medicines for the patients
- Patients buying meds from outside
- nurses keeping emergency medicine box with them
- all the suggestions related to medicine availability

This code will apply for all the health facilities - DH, MCWC, UHC, Private facility

---

Medicine supply_FRUSTRATION

- poor quality of medicines supplied
- Problems with the supply chain and their involvement in supplying the medicines
- Suggestion related to how to making the medicines supply properly

---

Indicator_Suggested New
All the suggested new indicators will be coded here

Newborn care_District Hospital

This code will include:

- all kinds of newborn care - delivering at the facility or coming after delivery from community or from other facility
-newborn death and illnesses
- what preparedness the facility has and what does not
- barriers in providing appropriate care
- non-complying with referral
- barriers felt from community like patients not wanting to comply with the referral at the higher level facility

(please note that some part of the discussion related to this issue may recoded again under specific provides (i.e. neonatologist) if that comes as a barrier to provide the newborn care)

Newborn care_Maternal and Child Welfare Centre

This code will include:

- all kinds of newborn care - delivering at the facility or coming after delivery from community or from other facility
-newborn death and illnesses
- what preparedness the facility has and what does not
- barriers in providing appropriate care
- non-complying with referral
- barriers felt from community like patients not wanting to comply with the referral at the higher level facility

(please note that some part of the discussion related to this issue may recoded again under specific provides (i.e. neonatologist) if that comes as a barrier to provide the newborn care)

Newborn care_Private Facility

This code will include services at private facilities:

- all kinds of newborn care - delivering at the facility or coming after delivery from community or from other facility
-newborn death and illnesses
- what preparedness the facility has and what does not
- barriers in providing appropriate care
- non-complying with referral
- barriers felt from community like patients not wanting to comply with the referral at the higher level facility

(please note that some part of the discussion related to this issue may recoded again under specific provides (i.e. neonatologist) if that comes as a barrier to provide the newborn care)

Newborn care_Upazila Health Complex

Obs and maternal care_District Hospital

This code will include:

- rate of all kinds of delivery
- All kinds of delivery related services
- why certain delivery like forceps or VA are carried out or not carried out - what needs to be done?
- Painless delivery
- all kinds of obs. and maternal care
- ANC care information
- barriers in providing this care appropriately (please note that some part of the discussion related to this issue may recoded again under specific provides (ex. obs. gyn consultant) if that comes as a barrier to provide this care)
- barriers felt from community like patients not wanting to comply with the referral at the higher level facility

---

Obs and maternal care_Maternal and Child Welfare Centre

This code will include:

- rate of all kinds of delivery
- All kinds of delivery related services
- why certain delivery like forceps or VA are carried out or not carried out - what needs to be done?

- Painless delivery
- all kinds of obs. and maternal care
- ANC care information
- barriers in providing this care appropriately (please note that some part of the discussion related to this issue may recoded again under specific provides (ex. obs. gyn consultant) if that comes as a barrier to provide this care)
- barriers felt from community like patients not wanting to comply with the referral at the higher level facility

---

Obs and maternal care_Private facility

This code will include:

- rate of all kinds of delivery
- All kinds of delivery related services
- why certain delivery like forceps or VA are carried out or not carried out - what needs to be done?
- Painless delivery
- all kinds of obs. and maternal care
- ANC care information
- barriers in providing this care appropriately (please note that some part of the discussion related to this issue may recoded again under specific provides (ex. obs. gyn consultant) if that comes as a barrier to provide this care)
- barriers felt from community like patients not wanting to comply with the referral at the higher level facility

---

Obs and maternal care_Upazila Health Complex

This code will include:
- rate of all kinds of delivery
- All kinds of delivery related services
- why certain delivery like forceps or VA are carried out or not carried out - what needs to be done?
- Painless delivery
- all kinds of obs and maternal care
- ANC care information
- barriers in providing this appropriate care
- barriers felt from community like patients not wanting to comply with the referral at the higher level facility

Please note that, when discussion related to not providing such care appropriately will be concerning to any particular physicians, that piece will be coded by that provider's code. However, if the discussion is generally talking about HR, and not mentioning about the unavailability of any particular provided - then this will be coded here

---

Overall situation_Public facility_FRUSTRATION

- Overflow of patient flow
- overall discussion about unavailability of physicians and staff for providing service (when a specific physician's name will be mentioned, then that will be recoded for that particular provider)
- Overall discussion about other problems for providing services
- Cases with severe complications after having delivery (either with risky mother and risky newborn) coming to Public facilities putting public facility in risk

---

Private facilities in the community

- No. of private facilities in the communitiy
- Type of private facilities - profit/noon profit
- Services provided in the facility

Private facility_C-section_CONCERN

Discussion related to concern regarding number of c-section in the private facility, and all the other information related to c-section will be coded here.

- How to monitor them to regulate the c-section.
- The reporting of Private Facilities regarding their C-Section data to HMIS
- Issues related to providing data on other services to HMIS, why they are not doing it - who provides and who does not and why etc.
- How to make them accountable and make them to provide data to HMIS - motivation, training, taking legal actions, making them accountable, monitoring them etc

---

Private facility_EmONC framework

- What are the suggested indicator for private facilities
- What are the suggested signal function for private facility
- Discussion and suggestion whether Private facility should be basic or comprehensive or not

- Requirement for Private Facilities to be considered under EmONC facility
- Discussion and suggestion whether Private facility should be under EmONC framework or not
- Any infrastructural setup of the private facility which play a role as a barrier to provider MNH care will come here (space. instrument, medicines, providers, neonatal care instrument, blood bank/transfusion, test and examination)

Note that some part of this coded section may be recoded for 'Private Facility_C-section'

Promoting normal delivery

Recruitment issues_FRUSTRATION

- Vacant posts
- Suggested necessary or required posts creation
- recruiting providers leaving the station and the post remaining vacant
- unnecessary posts or necessary posts which are not in the algorithm

(Sometimes some of this coded section may be recoded as the 'Frustration_Central and local lebel management'")

Referrals

All kinds of referral information will be coded here

- Referral from private facility
- referral from other public facility
- problems related to referral (information related to ambulance will be coded here as well as recoded for 'Infrastructure_Ambulance'
- Community's non-cooperation to referral

Monitoring-mentoring-reporting_Public facility

- Public facility reporting to HMIS and problems surrounding this
- Monitoring the public health facilities
- monitoring any staff's activity at a particular facility
- monitoring the data
- monitoring team coming from outside
- Problems with monitoring
- Suggestion to strengthen monitoring

Responsibility_Civil Society and others

- Responsibilities of civil society. local govt. bodies, community leaders and so on to ensure the services at the facility
- Advocasy by the political reprentative bodies

Role of brokers_CONCERN

Role of Hospital committee_FRUSTRATION

- Expected role of hospital committee,
- What this committee do and what they are supposed to do.
- Is this functional and if not why not
- Suggestion related to how to make them functional

Signal Function_Assisted or instrumental vaginal delivery

- Provider's perceptions and experience
- client's demand
- facility readiness
- training of the providers

SF_Blood Transfusion

SF_Manual removal of the placenta

SF_Neonatal resuscitation

SF New

SF_Obstetric surgery

SF_Parental Oxytocin

SF_Parenteral antibiotics

SF_Parenteral anticonvulsants

SF_Removal of retained products

Strengthening ANC

Slider

Sub-centre

Any discussion related to Sub-Centre

Suggestion_ Research and advocacy

Suggestions related to further research and assessment of the health facility in terms of infrastructure, HR and related suggested advocacy will be coded here

---

Support for poor

- Suggestion regarding making the health facility poor friendly
- Role of social welfare ministry
- corporate social responsibility of the private facility
- Any other support to the poor

Technology

This code will incorporate discussion related to availability of technology – different diagnostic machines and equipment and frustration related to this issues

---

Training

All kinds of training EXCEPT EOC training will be coded here

- However, when training for the Private facility providers will be discussed as part of motivating them, that will be coded under 'Private facility_Reporting and Monitoring'

Unavailability of providers_FRUSTRATION

- There are codes for every providers and staff where we will code particularly for a type of provider's/staff's un/availability, vacant posts, required posts and so on. However, this code will incorporate those discussions when any informants expressed frustration related to overall human resource situation or unavailability of any particular physicians/staff.
- suggestion for retaining the physicians

ALSO, Human Resource problems, other problems for the Civil surgeon and her/his office in carrying out her/his role and responsibilities for the functionality of the health facilities

---

Malawi

Number	Parent Node	Sub/Child Nodes	Grand child node	Applied to	Description	When to use/not use	Example	Nvivo Code
	**What works well/Facilitators**			All groups	Use works-well node on all discussion related to issues that are working well and help midwives to effectively manage emergency obsterict signal functions at their work stations or while in transit of patient referral.			Works well
		Staff capacity		All groups	Use staff capacity sub node on all general text where participants said midwives have what it takes to perfom any of the procedures/ signal functions related to management of emergency obstetric problems which is not specific to knowledge or skills			Staff capacity
			Knowledge	All groups	Use knowledge sub node on all text where participants said midwives have knowledge to perfom any of the procedures/ signal functions related to management of emergency obstetric problems			Knowledge
			Skills	All groups	Use skills sub node on all text where participants said midwives have skills or practical no how to perfom any of the procedures/ signal functions related to management of emergency obstetric problems			Skills
		Availability of resources		All groups	Use availability of resources node to code all texts where participants reported availability of general resources (both human and material) as something that is working well to aid the work of midwives at their work stations	Do not use this code when particioants pecify resouces such as avaiability of staff,		Resources-availabile
			Availability of human resources	All groups	Use availability of human resources sub node to code all texts where participants reported availability of staff/midwives as something that is working well to aid the work of midwives at their work stations			Human resources_available
			Availability of material resources	All groups	Use availability of material resources sub node to code all texts where participants reported availability of materials reources as something that is working well to aid the work of midwives at their work stations		Availabilty ofcatheters	Material resources_available
			Availability of drugs	All groups	Use availability of drugs sub node to code all texts where participants reported that required drugs are availabe as something that is working well to aid the work of midwives at their work stations			Drugs_available
			Availability of equipment	All groups	Use availability of equipment sub node to code all texts where participants reported that required equipement are availabe as something that is working well to aid the work of midwives at their work stations		We have vacuum extraction machines	Equipemnt_available
			Availability of paper	All groups	Use availability of paper sub node to code all texts where participants reported that required paper based resources are availabe as something that is working well to aid the work of midwives at their work stations		Availability of referral forms, partographs	Paper-available
			Availability of lab materials	All groups	Use availability of lab materials sub node to code all texts where participants reported that required diagnostic materials are availabe as something that is working well to aid the work of midwives at their work stations			Lab materials_available
		Relationship		All groups	Use relationship sub node to code all text where participants reported that there is good retionship among themselves and those at other facilities			Relationship
			Team work	All groups	Use team work sub node to code all text where participants reported that there isteam work among themselves and those at other facilities or or staff help each other to manage pateints with emergecy obstetric complication			Team work
			Good communication	All groups	Usegood communication sub node to code all text where participants reported that there is good communication or means of communication between BEMONC and CEMONC facilities			Good communication
		Availability of transport		All groups	Use availability of transport node to code all text where particioants reported that transport or means of transportation is available to transport patients from BEMONC to CEMONC facilities			Transport_available
		Availability of policies and guidelines		All groups	Use availability of policies and guidelines to code all text where participants reported that necessary policies and guidelines are available that helps midwives to manage emergency obstetrict signal functions at teir work stations			Policies_available
10	**Challenges/Barriers**			All groups	Use challenges node on all discussions related to challenges/problems that are available that affect the work of midwives to effectively manage emergency obsterict signal functions at their work station or while in transit of patient refreal.			Challenges
		Transport		All groups	Use transport sub node on all discussions related to transport challenges/problems that may delay acute cases to be transported from BEMONC to CEMONC sites in time.			Transport
			No stationed ambulance		Use no stationed ambulance sub node on all discussions related to non availability of ambulance stationed at the BEMONC site as a transport challengesproblemsthat may delay acute cases to be transported from BEMONC to CEMONC sites in time.		Unavailability of stattioned ambulance for referal	Ambulance_Nonstationed
			Fuel problems		Use fuel problem sub node on all discussions related to lack of fuel for ambulances as a contributing factor to the non availability of ambulances to transport acute cases from BEMONC to CEMONC sites in time.			Ambulance_Fuel
			Shortage of ambualnces		Use shortage of ambualnce sub node on all discussions related toshared ambualances that serve many facilities as a contributing factor to the non availability or delayed of ambulances to transport acute cases from BEMONC to CEMONC sites in time.			Ambulance_shortages

			Ambulances asigned to non-referal duties		Use Ambulances asigned to non-referal duties sub node on all discussions related tonon availability of ambualances becuase the ambualnce has been asigned to other duties other patients related as a contributing factors to the non availability or delayed of ambulances to transport acute cases from BEMONC to CEMONC sites in time.			Ambulance_nonreferral duties
		Lack of confidence		All NMs and MNT	Use lack of confidence sub node on all text where participants said midwives lack confidence or have confidence issues e.g. uncertainity or lacking confidence in the procedure other than failing to manage any of the emergency obstretric signal funtions due to fear, low self esteem and feeling less empowered			Confidence
			fear	All NMs and MNT	Use fear sub node on all text where participants said midwives fear to perfom any of the procedures/ signal functions related to management of any emergency obstetric problem			fear
			low self esteem	All NMs and MNT	Uselow self esteem sub node on all text where participants said midwives feels less empowered especially in the eyes of other professionals or have low self esteem to perfom any of the procedures/ signal functions related to management of any emergency obstetric problem			Low_self_esteem
		Lack of resources		All groups	Use lack of resources node to code all text where participants reported that the work of midwives is not done or not completely done due to absence of something other than drug, lab materials andequipment			Lack of resources
			Stockouts-drugs	All groups	Use stockout_drugs sub node to code all texts where participants reported that the work of midwives is not done or not completely done due drug stock outs e.g lack of oxytocin			drug_stock_outs
			Stockout_lab materials	All groups	Use Stockout-lab materials sub node to code all texts where participants reported that the work of midwives is not done or not completely done due stock outs of lab materials e.g. lack of urine dip sticks			lab_stock_outs
			Stockout_paper	All groups	Use Stockout_paper sub node on all text where participants discuss about lack of paper related resources that is critical to the work of midwives e.g. lack of patographs			Paper
			Equipment	All groups	Use equipment sub node to code all texts where participants reported that the work of midwives is not done or not completely done due lack of eipments that are vital for their job e.g lack vacuum extraction machine, lack of of battereis for Bp machines and lak of Bp cuffs etc			Equipment
		Lack of capacity			Use lack of capacity sub node on all general text where participants said midwives lacks knowledge or skills to perfom any of the procedures/ signal functions related to management of emergency obstetric problems			Lack of Capacity
			Lack of theoretical knowledge		Use lack of knwodge sub node on all l text where participants said midwives fails to perfom any of the procedures/ signal functions related to management of emergency obstetric problems due to lack or limited knowledge			Lack of Knowledge
			Lack of skills		Use lack of skills sub node on all text where participants said midwives fails to perfom any of the procedures/ signal functions related to management of emergency obstetric problems due to lack of or limited skills			lack of skills
		Lack of incentives			Use lack of incentives node to code all texts where participants reported that they fail to do or effectively do all parat of their work due to no incentives to motivate them.		Midwives from BEMONC facilities not escorting participant to CEMONC facilities because when they do that, they are not given accomodation to spend a night on	Incentives
		Negative attitudes		All groups	Use negative attitudes node to code all text where participants reported that health workers' negative attitudes had a negative impact on or affected midwifery care			Attitudes
		Systems challenges		All groups	Use systems challenge node to code all texts where participants report systems' level challenges other than policy related		bad roads hindering patients to be transported to the district hospital.	Systems

			Policies and guidelines	All groups	Use policies and gudelines sub node to code all texts where participants reported lack of policies or guidelines affecting their work or where midwifes are restricted to do their work or have necessary resources due to plocy restrictions		Challenges that arise from the Ministry of health head quarters and its related policies	Policies
			Communication challenges	All groups	Use communication sub node to code all texts where participants reported communication problems between BEMONC and CEMONC facilities		Lack of facility phones and airtime by BEMONC facilities; no feedback after referening patient; not network	Communication
			Staffing	All groups	Use staffing sub node to code all text where participants describes challenges related to staffing that affect midwives to effectively deliver care to their patients		Shortage of staff; scheduling problems; having non trained staff at facility	Staffing
		Patient challenges			Use patient challenes sub node to code all text where participants reported specific challenges arising from patients that affects the work of midwives		Patients failing to disclose full history; patients refusing refrerral	Patient_Challenges
11	**Suggested solution**			All groups	Use suggected solution node to all discussion participants talk about the proposed solutions to the identified Challenges or barriers			Solutions
		Capacity building						
			Mentorships					
			Supervison					
			Trainings					
			Support					
		Policy and advocancy issues						
			Upgarade rural hospitals					
			Policies and guidelines to be updated					
			Deploy more skilled staff at BEMOC sites					
			Provision of airtime					
		Need for reliable ambulance					Stationed ambulance	
	**Good quotes**			All groups	Use good quotes node related to all nodes through out coding			
		Specifc node						
		Specifc node						
		Specifc node						
		Specifc node						

COLLECTE DES DONNÉES (GROUPES DE DISCUSSIONS)

THEMES	Nurse midwives	Medical doctors 1	Managers 1	Medical doctors 2	Managers 2	Coordinators
**MAPPING DE LA SITUATION ACTUELLE DES SONU ET SYSTEME IDEAL SOUHAITE**<br><br>1. Fonctionnement actuel des structures SONU	**Ressources Humaines** +++ (priorisation):<br>- <u>pléthore</u> impactant sur l'organisation et la gestion des services (SONUC/CHU et hôpitaux nationaux)<br>- <u>Insuffisance</u> au niveau des SONUB/CS impactant la qualité des services, surcharge de travail…<br>- <u>Profil des RH/taches</u> : non adaptation y compris pour le personnel d'appui impactant par exemple sur la disponibilité des équipements (détérioration)…<br>- <u>Compétences pour les SONU</u> notamment des sages femmes depuis la formation de base avec la pléthore d'écoles de formation. Ces insuffisances dans la pratique sont plus ou moins supplées par le coaching sur sites.<br>- Faiblesse de la <u>prise en charge du nouveau-né</u> faute de personnel dédié	**Ressources Humaines** +++ (priorisation): LE FACTEUR HUMAIN<br>-<u>Burn out du personnel</u> du fait de (i)l'insuffisance des RH dans les EPS périphériques entraînant une surcharge de travail (1 sage femme de garde pour la gestion des soins à la mère, et au nouveau né, taches administratives/registres, et surveillance des autres services de la maternité) avec l'impact sur la qualité des SONU et la continuité des soins. Situation, aggravée par la mauvaise répartition des tâches.<br>(ii) l'état dans lequel les patientes sont reçues/retards,	**Ressources Humaines**:<br>-<u>Gestion des ressources humaines</u>: (i)pbme du recrutement des RH non basé sur le savoir être (volet important non pris en compte, ainsi que les aspirations et attentes du personnel avant recrutement/affectation); (ii) pbme de motivation et reconnaissance positive des prestataires; (iii) pbme d'équité dans le traitement du personnel pris en charge par les communautés et celui pris en charge par l'Etat; (iv) absence d'appui psychologique des RH notamment celles en milieu décentralisé.	**Faible prise en compte du nouveau né dans les SONU** malgré des initiatives (coin nouveau né, fonctions SONU nouveau né ajoutés au Sénégal, introduction SMK…). Le bas blesse en salle d'accouchement (*"on dirait que les SONU s'arrêtent juste à l'accouchement")* faute de prestataire dédié au nouveau né (Pédiatre d'un HR);<br><br>**Défi des RH**:<br>(i) Pbme de compétences dans la prise en charge du NNé.<br>(ii) insuffisance	**Pbme de disponibilité des médicaments d'urgence et des équipements clés:** du fait des difficultés de recouvrement des trousses d'urgence, de la gestion des stocks (besoins non satisfaits au niveau des pharmacies de district et régionales), mais aussi des problèmes de gouvernance dans la gestion des stocks au niveau des structures de santé (salles d'accouchement).<br>Les problèmes de disponibilité d'équipements fonctionnels sont dûs à la faiblesse de la maintenance (non considérée à la place importante qu'elle doit avoir) faute de ligne budgétaire et du non renouvellement/	**Problèmes de disponibilité du sang** nécessitant la mise en place de Centres régionaux de transfusion sanguine, et de dépôts de sang dans les structures faisant la transfusion (toutes les structures avec blocs de sang).<br><br>**Problèmes de la référence:** disponibilité des ambulances et carburant (PS ne disposent pas de ligne budgétaire pour la référence alors que ce sont eux

THEMES	Nurse midwives	Medical doctors 1	Managers 1	Medical doctors 2	Managers 2	Coordinators
	au nouveau-né dans les salles d'accouchement (en particulier dans les SONUB, disponibilité d'infirmières néonat dans certains hôpitaux), ou faute de répartition des tâches.<br>- <u>Besoins en sages femmes</u> : 3/30 accouchements par mois OK mais pas encore appliqué dans tous les SONUB d'ou la surcharge de travail.<br><br>**Médicaments d'urgence** +++ (priorisation): Problème de financement pour la disponibilité permanente des médicaments clés pour les SONU : objectifs/logiques divergents entre prestataires et administratifs ; Différentes stratégies mises en œuvre (facturation, caution, remboursement des produits par les patientes…). Nécessité de	(iii) la non disponibilité des médicaments (*"J'ai un amour ardent pour une salle d'hospitalisation bien équipée pour une meilleure prise en charge des patientes"*) **,<br>(iv) impact psychologique des décès maternels sur le personnel (*"il m'arrive de perdre une malade dans des conditions difficiles et ça fait mal!"* **).<br>-<u>Esprit d'équipe</u> comme facteur clé pour la performance et l'organisation des services ("*les relations entre les prestataires permet de gagner beaucoup de temps*") **<br>-<u>Comportement du personnel/ empathie</u> comme élément de qualité des SONU pour des soins respectant la dignité	*Déclaration d'un prestataire en zone décentralisé à son superviseur "Dr je sais que je peux faire le travail, mais j'ai besoin d'un soutien psychologique"*; (v) gestion des RH non décentralisée; (vi) spécialistes ne sont pas doublés dans les structures périphériques (gynécologues, pédiatres, anesthésistes avec risque de burn out et abandon de postes); (vii) Accent est plus mis dans les structures sur le savoir faire que sur le savoir être;<br>-<u>Importance du travail en équipe</u>: permet d'être performant dans la durée et permet de suppléer aux conditions de travail difficiles;	de RH qualifiées dans les structures périphériques y compris dans certains hôpitaux régionaux; (iii) les prestataires non formés ne s'occupent pas du nouveau né (*"les prestataires : sages femmes, médecins, ont peur du Nné"*); Cependant dans certains hôpitaux il existe des initiatives consistant à mettre en place des équipes de prestataires formées et dédiés au nouveau né dans la salle d'accouchement au niveau du coin NN voire des unités de néonatologie au	amortissement des équipements.<br>Les coins du nouveau né ne sont pas toujours aux normes (non disponibilité du matériel de réanimation NN et personnel insuffisant).<br>Pbme de responsabilisation de la gestion du coin du nouveau né non rattaché à la pédiatrie.<br>Mauvaise distribution/planificati on des équipements qui fait que certaines structures disposent de matériel alors qu'ils n'ont les RH pour les faire fonctionner et d'autres ne peuvent fonctionner faute de ce même type d'équipements alors que le personnel est disponible.<br>**Pbme des RH:**<br>(i) pbme de compétences pour l'offre de SONU (ex: la ventouse), certains	qui réfèrent vers le niveau supérieur).<br>**Faible implication des collectivités locales dans la gestion des ambulances**. Cependant des expériences positives à ** et ** dans l'implication des préfets pour la dotation du carburant ont été rapportées.<br><br>**Insuffisance de RH qualifiées en périphérie**. Au niveau de la région de ** par contre il y a des RH mais d'un âge avancé donc peu investi dans la gestion des urgences

THEMES	Nurse midwives	Medical doctors 1	Managers 1	Medical doctors 2	Managers 2	Coordinators
	revoir le mécanisme de financement de la santé…<br><br>**Référence** +++ (priorisation):<br>-Insuffisance de la disponibilité des ambulances y compris ambulance médicalisé impactant les délais de prise en charge ;<br><br>- Non respect des règles de référence (communication vers la structure de référence, outils/fiche de référence, moyens de transport non adaptés, absence de mise en conditions avant la référence …). Impact positif des séances d'audit des décès maternels et les réunions de coordination des districts impliquant les SONUC/hôpitaux pour le feedback SONUC/SONUB.<br>Impact positif du SAMU dans les régions qui en disposent.	humaine *"une femme qui accouche doit être écoutée et accompagnée, donner une vie c'est sacré. **".*<br>- <u>La satisfaction du personnel</u> est lié à (i) l'environnement de travail: disponibilité des médicaments et équipements, aux conditions et ambiance de travail, mais aussi (ii) le niveau de rémunération, (iii) la formation continue, (iv) l'attente d'une affectation/sentiment d'être oublié dans les structures périphériques sans perspectives d'affectation.<br>-<u>Déperdition du staff</u> bien formé dans les hôpitaux quittant pour d'autres postes voire d'autres départements (ex: éducation), et turn over faute de	**Intrants:**<br><u>Problématique de l'accès au sang</u>:<br>(i)pbme de dons volontaires avec pérennisation des donneurs, (ii) pbme de logistique et intrants pour la collecte, (iii) absence de banques de sang régionales et de dépôts secondaires de sang dans les SONUC (**) *"on ne peut pas avoir un réseau SONU sans une autonomisation de la gestion du sang"*;<br><u>Problème du financement des médicaments et produits d'urgence</u>: essentiellement dû au non-respect de la clé de répartition des bénéfices générés par la vente des médicaments dans le cadre de l'IB, et à l'abandon du monitoring des SSP	sein des maternités.<br>**Équipements et médicaments:**<br>(i) coins nouveaux nés non fonctionnels (table de réanimation aux normes) surtout dans les structures périphériques; (ii) Pbme de disponibilité de l'Oxygène et des médicaments d'urgence pour le Nné<br><br>**Référence des NNés malades ou de petit poid**s:<br>Outil de liaison mis en place pour améliorer les références non disponibles au niveau périphérique<br><br>**Relations conflictuelles**	matériels ne sont pas utilisés faute de compétences (ex de pousse-seringues dans un EPS), (ii) insuffisance de la délégation de tâches surtout pour les blocs SONU non fonctionnels faute d'une partie de l'équipe (ex: pour les anesthésistes, formation de base trop longue versus formation des médecins compétents SOU); (iii) pbme de rétention du personnel qualifié (faible implication des collectivités locales); (iv) équipes SOU non doublées et impact psychologique/épuisement; (v) dans les EPS certains spécialistes n'assurent pas la garde (ex: un seul pédiatre dans un EPS);<br><br>Les audits des décès n'ont pas l'impact attendu car c'est	obstétricales et néonatales.<br><br>**DHIS2 et réseau SONU**:<br>Il est la seule source de référence pour le calcul des indicateurs SONU. Cependant le calcul des indicateurs devrait être amélioré dans le rapport standard (les coordinatrices utilisent des données saisies pour calculer elles-mêmes les indicateurs).

THEMES	Nurse midwives	Medical doctors 1	Managers 1	Medical doctors 2	Managers 2	Coordinators
		véritable stratégie de rétention du personnel.	qui permettait de suivre la gestion des produits et leur financement.<br><br>Absence de plan d'amortissement du matériel: Permettrait de planifier le renouvellement avant détérioration ou obsolescence.<br><br>Fonctionnalité des blocs SONU:Absence d'indicateur de suivi de la fonctionnalité des blocs SONU	**entre les prestataires et la population:** avec culpabilisation des prestataires par les population créant un sentiment de désarroi chez les prestataires	surtout la recherche des boucs émissaires plutôt que celle des solutions pour éviter que de telles situations se reproduisent, et aussi l'attitude de défensive et de non prise de responsabilité de la part des prestataires. D'où l'importance de privilégier les "Near miss" plutôt que les revues des décès.	
		SONUB et SONUC bonne classification actuelle				La classification SONUB et SONUC OK mais possibilité d'avoir un niveau intermédiaire qui assure la fonction transfusion sanguine (maternités des

THEMES	Nurse midwives	Medical doctors 1	Managers 1	Medical doctors 2	Managers 2	Coordinators
						CS avec médecin).
		Impact positif du réseau SONU : moins de références et moins de décès maternels lorsque les SONUB fonctionnent (renforcement du plateau technique, formation des SFs…)	**Réseau SONU actuel important** notamment pour le renforcement des capacités des SONUB désignés (*"certaines structures demandent à être incluses dans le réseau")*, le monitoring, avec renforcement de la collaboration entre structures du réseau, mais défi de pérenniser la fonctionnalité 24h/24.	**Réseau SONU** peu connu des pédiatres en dehors de ceux qui sont impliqués dans le monitoring.	**Réseau SONU** se heurte aux pbmes de référence faute de régulation surtout à **, aux difficultés pour les lignes téléphoniques (pas de dispositif de mise en réseau des structures de santé pour éviter le coût des appels téléphoniques);	**Le réseau SONU** a des points faibles et des points à améliorer. Il permet de (i) suivre la fonctionnalité des structures SONU en particulier les blocs SONU, (ii) permet le respect des protocoles de prise en charge (ex: administration de la tri antibiothérapie pour les infections), (iii) de renforcer les compétences des prestataires lorsque le besoin est identifié dans le cadre du

THEMES	Nurse midwives	Medical doctors 1	Managers 1	Medical doctors 2	Managers 2	Coordinators
						monitoring, (iv) l'amélioration de la disponibilité des médicaments (ex: réorganisation de la gestion des médicaments entre la PRA et les maternités, mise en place des conditions pour le respect de la chaîne de froid pour le syntocinon dans des maternités). Cependant, il y a **une faible appropriation par les MCDs,** les communautés faute de restitution communautaires (CDS et communauté, autorités

THEMES	Nurse midwives	Medical doctors 1	Managers 1	Medical doctors 2	Managers 2	Coordinators
						locales/maires, préfets, gouverneurs…), **et surtout problème de suivi et de mise en œuvre des recommandations du monitoring.** Une des raisons de la faible appropriation par les districts est le schéma utilisé lors de l'introduction du monitoring qui a plus impliqué les coordinatrices SR et les MCR plutôt que les districts.<br><br>Une des conséquences de la non implication des districts est le non respect de

THEMES	Nurse midwives	Medical doctors 1	Managers 1	Medical doctors 2	Managers 2	Coordinators
						l'affectation des SFE dans les structures du réseau pour respecter la normes établie (au moins 3 SFE pour fonctionnement 24/24 renforcé en fonction de l'activité obstétricale; et ceci malgré l'identification des besoins par le niveau régional (répartition des RH étant du ressort des districts) et les opportunités de recrutement existantes.<br><br>Une autre conséquence est l'insuffisante implication des districts dans la

THEMES	Nurse midwives	Medical doctors 1	Managers 1	Medical doctors 2	Managers 2	Coordinators
						gestion de la référence (carburant pour les ambulances), avec des expériences positives d'implications de MCD dans la dotation de carburant à partir de la dotation du carburant du fonctionnement du district, en collaboration avec les collectivités locales.<br><br>Le monitoring des SONU tel qu'il est conduit "ne permet pas de valoriser les efforts des structures pour rendre les SONU disponibles"

THEMES	Nurse midwives	Medical doctors 1	Managers 1	Medical doctors 2	Managers 2	Coordinators
						surtout des hôpitaux qui se sentent démotivés par le monitoring. Ceci est dû à la manière d’apprécier la disponibilité des fonctions SONU qui crée bcp de polémiques entre les acteurs du niveau opérationnel et ceux du niveau central (interprétation divergente de la manière d’évaluer la disponibilité des fonctions SONU). La gestion des médicaments est différente dans les hôpitaux et devrait être pris en compte.

**THEMES**	**Nurse midwives**	**Medical doctors 1**	**Managers 1**	**Medical doctors 2**	**Managers 2**	**Coordinators**
						Problème de régularité du monitoring faute de décentralisation du financement au niveau des régions.
2. Système idéal pour les SONU	Disponibilité d'ambulances médicalisés<br><br>Infrastructures modernes avec intégration de la maternité avec la pédiatrie (prise en charge mère-enfant), tables d'accouchements équipées avec éclairage et confort<br><br>Disponibilité de salles de réanimations bien équipées<br><br>Locaux propres avec personnel d'appui suffisant<br><br>Médicaments d'urgence accessibles (armoire d'urgence en salle	Importance de **l'entretien et du nettoyage des locau**x (prévention des infections)<br><br>R**égulation de la référence** impliquant par exemple le SAMU<br><br>**Fonctionnalité des ambulances médicalisées** (carburant) surtout au niveau des CSII qui même s'ils sont SONUC évacuent au niveau EPS notamment lorsque le plateau technique est défaillant (ex: non disponibilité du sang dans les CSII,	Organisation d'un système de rotation du personnel en particulier spécialistes dans les zones décentralisées;<br><br>Disponibilité de mécanismes d'écoute entre prestataires et superviseurs aux différents niveaux, et de mécanismes de soutien psychologique pour les prestataires;<br><br>Performance des structures doit être basée aussi sur le savoir être en plus du savoir faire.	- La nécessité de mettre en place un cadre intermédiaire (infirmier/assistant infirmier néonat) pour la prise en charge du nouveau-né en salle d'accouchement car *« les sages femmes ne peuvent pas s'occuper en même temps de la mère et du nouveau-né ».*<br>- La nécessité de mettre en place des unités de néonatologie légères en salle	-Les médicaments d'urgence doivent être subventionnés par l'Etat; Nécessité de développer des procédures de recouvrement des coûts des médicaments d'urgence dans les SONUC.<br>-Nécessité d'un feedback de la communauté<br>-Besoin de doubler les équipes des blocs SOU;<br>- le coaching in situ est un excellent moyen de renforcer les capacités des prestataires;<br>- les RH ont besoin d'assistance psychologique pour mieux gérer le stress…	Le monitoring des SONU devrait inclure la communauté (CDS et population), les collectivités locales et autorités administratives (préfets, sous préfets).<br><br>Le financement du monitoring devrait être décentralisé aux régions pour une meilleure appropriation et efficacité.

THEMES	Nurse midwives	Medical doctors 1	Managers 1	Medical doctors 2	Managers 2	Coordinators
	d'accouchement avec tous les médicaments clés)	EPSI, respirateur, coins NNé non fonctionnels…). **Organisation des services:** -Gestion du coin du nouveau-né qui doit être sous la responsabilité de la pédiatrie dans les hôpitaux. <br>-Communication entre gynécologues et pédiatres y compris avant la naissance (grossesses pathologiques)	Nécessité de mesurer la qualité de la gestion des RH par les managers à travers par ex: des entretiens avec tous les membres du personnel notamment avant de leur confier des responsabilités, les mutations… <br>Nécessité de favoriser les espaces d'écoute (type rencontres miroir entre prestataires) pour améliorer le climat de travail et la performance	d'accouchement gérés par les pédiatres et en lien avec les services de pédiatries (l'après accouchement est du ressort des pédiatres). Car selon les pédiatres « *après l'accouchement, le nouveau-né est un peu oublié en salle d'accouchement, on dirait que les SONU s'arrêtent juste à l'accouchement* !). - Tout personnel qualifié (médecin, infirmiers, sages femme, assistant infirmier) peut offrir des soins de qualité aux nouveaux nés à condition d'être formé à la prise	Les coins du NNé doivent être sous la dépendance de la pédiatrie en attendant la mise en place d'unités de néonatologie dans les maternités; -les districts doivent être impliqués dans les plans de formation avec une meilleure prise en compte de leurs besoins en formation (privilégier les formations sur site/coaching); - nécessité d'accompagner les blocs SOU pour leur ouverture ou réouverture (RH, équipement); -Pour améliorer la disponibilité du sang (en attendant la mise en place de centres régionaux de transfusion sanguine), proposition de mettre en place une flotte de motos basées au niveau des banques de	Nécessité de mettre en place des **Centres régionaux de transfusion sanguine** sous la responsabilité desquels seront placés des d**épôts de sang dans toutes les structures SONUC** <br>Mettre l'accent sur l'offre effective de SONUB dans les structures devant offrir des SONUC. <br>Les dialogues communautaires devraient être institutionnalisés et monitorés. Mettre à

THEMES	Nurse midwives	Medical doctors 1	Managers 1	Medical doctors 2	Managers 2	Coordinators
				en charge du nouveau-né.<br>- la généralisation des SMK dans toutes les structures où se font les accouchements.<br>- la continuité des soins d'urgence pendant les évacuations;<br>- Référence devrait se faire avec les outils de référence (fiche de liaison et dans des ambulances médicalisées).	sang pour approvisionner les structures à la demande et dans des délais les plus courts possible.<br>-Nécessité de disponibiliser des centres régionaux de transfusion sanguine en charge de l'approvisionnement et du contrôle qualité des dépôts de sang dans tous les SONUC.	contribution des compétences externes au système de santé (sociologues…). Utiliser les boîtes à idées et les émoticônes pour alimenter les dialogues communautaires afin d'analyser les causes de non satisfaction des usagers.<br><br>Nécessité de standardiser les coûts des examens complémentaires (bilan prénatal).<br><br>Améliorer le calcul des indicateurs dans le rapport

THEMES	Nurse midwives	Medical doctors 1	Managers 1	Medical doctors 2	Managers 2	Coordinators
						standard du DHIS2.<br><br>Introduire les indicateurs de qualité dans la supervision formative.<br><br>Améliorer la communication des prestataires en direction des communautés qui ont un besoin de mieux comprendre les soins qui leur sont offerts et y adhérer.
3.Qualité des SONU	**Communication** +++ (priorisation): accueil, communication avec la patiente et la famille pendant les soins…<br>Causeries dans les hôpitaux ne sont font plus.<br>Sensibilisation versus engagement	**Préalables pour une qualité des SONU:**<br>-plateau technique adéquat (structures bien équipées)<br>-référence à temps<br>-RH qualifiées disponibles<br>-Relations entre les membres de l'équipe	**Besoin d'un indicateur composite pour évaluer la qualité des SONU incluant:**<br>(i)Compétences des prestataires pour l'offre de SONU;	Qualité des SONU est liée à la disponibilité :<br>- des infrastructures adaptées<br>- de RH qualifiées (spécialistes)<br>- de médicaments en	**Qualité des SONU doit être appréhendée dans le cadre d'une approche qualité avec des normes, standards et procédures liées aux RH, aux équipements…**<br>Elle est liée à l'environnement de travail, et au plateau	La qualité dépend de plusieurs éléments:<br>(i) la disponibilité des RH qualifiées; (ii) la disponibilité des intrants et

THEMES	Nurse midwives	Medical doctors 1	Managers 1	Medical doctors 2	Managers 2	Coordinators
	communautaire à discuter.<br>Contenu du counseling durant les CPN à revoir (informations sur l'accouchement, la gestion de la douleur, plan d'accouchement et préparation à l'accouchement…)<br><br>**Disponibilité du plateau technique et le confort des patientes** comme élément de qualité<br><br>**Gestion de la douleur** : besoin existe mais pas de conditions pour l'accouchement sans douleur même dans les hôpitaux.<br><br>**Perception de la qualité par les prestataires** :<br>Mauvaise qualité : File d'attente<br><br>Bonne qualité :<br>-satisfaction des clientes (patientes avec le sourire, discussions entre patientes),	-accessibilité financière<br>-possibilité de faire le bilan sanguin durant la grossesse (échographie privilégiée au détriment du bilan par les femmes notamment en milieu rural)<br><br>**Perception de la qualité par les prestataires** :<br>- Résultat final doit être mère et nouveau né bien portants<br>- Satisfaction des usagers: cependant absence de mécanisme de mesure de cette satisfaction;<br>-baisse de la mortinatalité	(ii)Disponibilité de procédures claires et respectées par les prestataires (iii) Disponibilité d'intrants (iv) Satisfaction des usagers (v) Létalité par cause?	quantité suffisante, équipements fonctionnels, et médicaments d'urgence (Gardenal inj, MgSO4, Adrenaline, Caféine, Diazépam)<br>- de données de qualité<br>- d'une maintenance préventive des équipements<br>- un bon accueil<br><br>**Résultats de la qualité** est la satisfaction des prestataires et des usagers	technique permettant l'offre de services (préalable);<br>Les populations sont sensibles aux changements qualitatifs dans les structures, *"c'est pourquoi il est important d'améliorer l'environnement de travail, la qualité des équipements, le confort et la propreté des locaux".*<br><br>Elle se manifeste pour le prestataire par un "*sentiment de devoir accompli*." mais les prestataires "*doivent aussi savoir recevoir les feedbacks négatifs de la population, il faut qu'on les laisse s'exprimer y compris dans les réseaux sociaux…*" **.<br><br>*Même si les populations manifestent leur satisfaction, le*	équipements;<br>(iii) le respect des protocoles de prise en charge; (iv) le suivi des indicateurs et des recommandati ons conditionné aussi par la collecte des données de qualité (outils de gestion bien remplis).<br><br>Pour les usagers, la qualité dépend (i) du degré de satisfaction des usages; (ii) accessibilité géographique et financière; (iii) la disponibilité des médicaments; (iv) environnement

THEMES	Nurse midwives	Medical doctors 1	Managers 1	Medical doctors 2	Managers 2	Coordinators
	-empathie du personnel,<br>-satisfaction des prestataires (résultats de la prise en charge, bonne organisation des services avec une répartition des tâches)				*prestataire a un sentiment mitigé du fait des manquements qu'il constate dans ses conditions de travail*.	de travail; (v) acceuil; (vi) attitude respectueuse et empathique du personnel.
**INFORMATIONS NÉCESSAIRES POUR LA PERFORMANCE DES SONU**<br><br>1. Besoins d'information	**Informations sur la référence** :<br>- Mise en condition<br>- Intégration des données nécessaires/clés dans les outils de référence<br>**Informations sur le financement/gestion des médicaments**<br><br>**Informations sur la formation des SF à monitorer :**<br>- Prise en compte des fonctions SONU dans la formation de base<br>- Données sur les compétences/pratique des sages femmes (doivent être prises en compte dans	Besoins d'I**nformations sur les médicaments et équipements**:<br>-disponibilité permanente des médicaments<br>-disponibilité permanente et fonctionnalité des équipements<br><br>**Informations sur les compétences des prestataires:**<br>-compétences en communication avec les usagers: ex niveau de connaissance des usagers sur leur diagnostic, connaissance du personnel ayant assuré la prise en charge…	**Référence:** Nécessité d'indicateurs de référence par ex: (i) disponibilité d'ambulances médicalisées, gérées par une structure de coordination qui assure la régulation, (ii) qualité de référence analysant le nombre de femmes référées des SONUB aux SONUC et le devenir de ces femmes<br><br>Besoin pour les managers d'**indicateurs d'alerte** qui même si les conditions sont présentes permettent de mesurer le risque de	-morts nés frais (intra partum)<br>-morts nés dans les 24h<br>-Nnés asphyxiés<br>-Nnés de faible poids de naissance<br>-Nnés réanimés<br>-Proportion de NNés réanimés qui survivent/sauvés<br>-Proportion de Nnés réanimés référés (distinguer référés pour meilleure prise en charge et référés faute de place<br>-Nnés examinés avant la sortie<br>-NNés avec infections	-**monitoring de la disponibilité des médicaments, intrants et matériel clé**<br>-**monitoring des compétences** des prestataires (ex: à travers la supervision formative)<br>-% des **coins du NNé aux normes**<br>-**disponibilité des RH pour fonctionner 24h/24**;<br>-**disponibilité des dépôts de sang** dans les SONUC<br>-**Données sur la référence**: ex: nombre de référées/complications, cartographie de la référence (qui et où), références selon les normes (outils, conditionnement,	**Informations sur la communication entre prestataires et communauté:** préalable et que cette communication soit mise en oeuvre/intégrée dans les activités de routine et monitorée, suivie dans les réunions de coordination

THEMES	Nurse midwives	Medical doctors 1	Managers 1	Medical doctors 2	Managers 2	Coordinators
	l'évaluation/la certification des sages femmes	-compétences des RH sur l'offre de SONU : ex exploitation de l'évolution de la raison de non offre des fonctions SONU pour cause de compétences<br>- comportement des prestataires en direction des usagers ex: avis des usagers/enquêtes de satisfaction des usagers sur le comportement des prestataires<br>-conformité de l'offre de services par rapport aux normes:ex partogramme systématique et de qualité par les sages femmes<br>- disponibilité d'une formation continue obligatoire en particulier pour les gynécologues<br><br>**Informations sur la référence**:	changements négatifs de ces conditions (à définir: tels que niveau de stocks en plus de l'état du stock, idem pour les RH dont la disponibilité peut être bonne à un moment donné mais avec un risque de changement: congés, départs…) ;	-Nnés bénéficiant de SMK<br>- NNés décédés entre 0-7j<br>- NNés décédés entre 8-28j<br>- Nbre de RH qualifiées formées à la prise en charge du Nné<br>- Compétences des RH pour l'offre de fonctions NNé<br>-Disponibilité des équipements fonctionnels par niveau<br>- Disponibilité des médicaments d'urgence et consommables<br>-proportion des Nnés référés avec les outils (fiche de liaison)/Informations sur les conditions d'accouchement	délai), taux de contre référence documentée (par rapport aux référées)<br>-**Données sur la maintenance**: ex Disponibilité des RH dédiées à la maintenance, disponibilité du plan d'amortissement et des outils de suivi du matériel;<br>-	

THEMES	Nurse midwives	Medical doctors 1	Managers 1	Medical doctors 2	Managers 2	Coordinators
		-délais de référence effectif entre la décision de référer et l'arrivée dans la structure de référence;<br>-conformité de la référence par rapport aux normes (outils, mise en conditions, accompagnement médicalisé…)<br>-disponibilité d'un système de référence à temps: carburant, chauffeur…<br><br>**Monitoring de la disponibilité de l'offre:** ex Nombre de jours où la structure n'est pas fonctionnelle faute de RH, équipement, médicaments…<br><br>**Mesure de la qualité:**<br>-délais de prise en charge initiale et délai dans le respect				

THEMES	Nurse midwives	Medical doctors 1	Managers 1	Medical doctors 2	Managers 2	Coordinators
		des différentes étapes du protocole				
2. Aspects non cliniques des SONU à mesure/ prendre en compte/ monitorer	-Communication : accueil y compris par le personnel non médical (ex : sécurité et entretien)<br>-Empathie<br>-Indicateurs de management des structures<br>-Esprit d'équipe<br>-Organisation des services : répartition des tâches, Surcharge de travail (mesurable par le temps de présence, volume d'activités ??)<br>-Impact psychologique sur le personnel de santé (ex : impact des décès maternels)	Besoin d'informations sur la **satisfactions des prestataires**:<br>-satisfaction du niveau de rémunération<br>- satisfaction des conditions de travail<br>- supportabilité de la charge de travail<br>- nombre de mois/années d'attente pour une affectation<br>- nombre de mois/années avec volonté de quitter le poste actuel<br><br>**Besoin d'informations sur la satisfaction des usagers: -**enquêtes de satisfaction avant (CPN), après accouchement (ex: cartons verts, orange, rouge dans la structure), lors des CPON, et quelques	Nécessité de mesure le savoir être des prestataires<br><br>Nécessité d'avoir le feedback de la communauté sur les SONU à travers par ex: boîtes à idées dans les structures, enquêtes de satisfaction, bureaux des usagers…	Satisfaction des usagers<br><br>Satisfaction des prestataires	-**degré de satisfaction des prestataires** (éléments possibles: rémunération, prise en charge sociale, cadre de travail, disponibilité équipements et médicaments…);<br>-**Point de vue de la communauté** (par ex à travers des fora avec les femmes, conjoints, famille, belle familles, grands mères…)<br>-**degré de satisfaction des usagers**;	**Informations sur la communication entre prestataires et communauté:** préalable et que cette communication soit mise en oeuvre/intégrée dans les activités de routine et monitorée, suivie dans les réunions de coordination

THEMES	Nurse midwives	Medical doctors 1	Managers 1	Medical doctors 2	Managers 2	Coordinators
		mois après au niveau de la communauté.				

SYNTHESE DE L'ATELIER DE VALIDATION DES INSIGHTS ET ECHANGES SUR LES NOUVEAUX INDICATEURS PROPOSES

COMMUNAUTES (Mères de nouveaux-nés, Pères de familles)

SESSIONS	POINTS DE DISCUSSION	RESULTATS/RECOMMANDATIONS
Session 1<br>**Soins maternels centrés sur la personne**	Déterminer si le score de la **maternité centrée sur la personne (PCMC)** rend compte des constructions qui sont importantes pour les femmes et les hommes.<br>Identifier les **sources et les canaux de confiance** pour recueillir ces informations de façon routinière.	
	A- Discussion sur les domaines du PCMC<br><br>• *Respect et dignité*<br>- Suivre et respecter le rang d'arrivée pour obtenir un soin<br>- Tenir compte de l'état du malade et de l'urgence (permet d'éviter de demander à la femme de faire des vas et viens pour acheter le ticket<br>- Éviter les violences verbales et physiques devant les « pairs »<br>- Reconnaitre et donner la place qu'il faut aux maris et accompagnants comme « partenaires » et « aidants »<br>- Expression et attitude du soignant á l'égard des hommes<br>- Infrastructures qui valorisent l'intimité de la femme<br>-<br>• *Communication et autonomie*<br>- Apaiser par la parole, dans un langage commun<br>- S'enquérir de sa situation et partager des informations sur son état<br>- Ne pas faire que des reproches,<br>- Dialoguer, Donner des explications, rassurer	NB : **RESPECT est la composante la plus importante pour les hommes et femmes,** car s'il y a respect, cela influence le mode de communication, la manière de prendre soin d'eux, de pratiquer un soin de soutien tout en accordant la place qu'il faut aux hommes et aux accompagnants

SESSIONS	POINTS DE DISCUSSION	RESULTATS/RECOMMANDATIONS
	- Donner des indications, recommandations sur la conduite à tenir<br>-<br>• *Soins de soutien*<br>- Patience dans la prise en charge (prendre son temps pour éviter la césarienne)<br>- Compassion, avoir pitié<br>- Identifier et satisfaire les besoins de la femme et de ses accompagnants<br>- Accueil avec assistance, discussions et conseils<br>- Rassurer le malade pour diminuer la peur et l'inquiétude<br>- Accompagner jusqu'au bout, disponibilité, se mettre à disposition des malades et de leurs accompagnants<br>- S'enquérir de la situation<br><br>***B- Discussion sur les sources de confiance***<br>• ***Pour les hommes***<br>- Femme épouse : confidente, compagnon<br>- Mères : responsables pour assister leur belle-fille<br>- Frères<br><br>• ***Pour les femmes***<br>- Mari : confidente, compagnon<br>- Mère de la femme : assistance, conseils<br>- Belle-mère : assistance, conseils<br>- Sage-femme : confidente	Logique de couple dans la gestion et la partage d'informations relatives aux expériences de soins<br><br>Les hommes sont de plus en plus visibles dans la fréquentation des services de santé maternelle et néo-natale (CPN, REFERENCES, ACCOUCHEMENTS), aussi bien en zone urbaine que rurale<br><br>Les mères des femmes mariées gardent encore une place importante dans le cercle de confiance (aux cotes des belles-mères) en raison de leur apport en conseils et assistance pouvant rassurer les femmes
**Session 2 :**		

SESSIONS	POINTS DE DISCUSSION	RESULTATS/RECOMMANDATIONS
**Préparation à la référence**	Déterminer si les composantes de l'indicateur d'aiguillage proposé rendent compte des constructions qui sont importantes pour les femmes et les hommes.<br><br>Identifier ce qui constitue **une référence " centrée sur la famille ".**	
	• **La préparation au transport**<br>- Problèmes de disponibilité d'ambulances<br>- Retards dans la mobilisation du chauffeur, souvent pas en position de gestion d'une urgence<br>- Payer pour le carburant avant d'organiser la référence (25000 ou 18250 FCFA).<br>- Absence de réserve de carburant qui rend difficile la référence la nuit dans les zones rurales (lorsque les stations d'essence ont fermé)<br>- Payer la location d'un véhicule coute beaucoup plus cher que le coût demandé pour l'ambulance<br>- Souvent on ne prépare pas les ambulances sur le plan technique, ce qui occasionne des pannes en cours de route (crevaisons, batteries), ce qui allonge<br>- Accompagnants ayant besoin de temps pour mobiliser l'argent, ce qui retarde la mise en œuvre de la référence<br>- On ne donne pas aux accompagnants le temps de réagir et se préparer pour la référence<br>-<br>• **les soins pendant le transport**<br>- présence de l'assistante de la sage-femme qui s'occupe de surveiller les perfusions	Un grand besoin d'une prise en charge réactive qui anticipe et se prépare à organiser la référence, dans le respect de la dignité<br><br>Une faible dimension de soutien dans l'organisation de la référence : exigence de payer l'argent pour déclencher la préparation et mobilisation de l'ambulance<br><br>Penser d'abord à la douleur, à l'urgence avant de penser à l'argent<br><br>une séparation qui ne rassure pas et ne permet pas de profiter de l'aide, de l'accompagnement et du soutien qu'ils peuvent apporter auprès des assistantes<br><br>Pour une référence centrée sur la famille, qui sollicite les accompagnants comme des « aidants »

SESSIONS	POINTS DE DISCUSSION	RESULTATS/RECOMMANDATIONS
	- soin très technique, plus focalisé sur la perfusion que l'assistance et l'accompagnement de la femme pour gérer la peur et l'inquiétude<br>- pas de respect de la douleur (assistante souvent au téléphone)<br>- faible communication pour rassurer et assister la femme, une faible promptitude de la communication<br>- place du mari ou des accompagnants à l'avant du véhicule est mal vécue : cela ne les rassure pas et ne respecte pas leur place dans un système de soins centre sur la famille<br>-<br>• **l'accessibilité financière de la référence**<br>- Coût et modalités de paiement sont une vraie contrainte pour les évacuations d'urgence<br>- Expérience de beaucoup de ruptures de médicaments dans les services publics de sante qui obligent à se tourner vers les pharmacies, ce qui alourdit le coût financier<br>- Cherté du coût des soins de référence laisse les familles avec beaucoup de dettes contractées<br>- Faible contribution de l'assistance sociale<br><br>• **soins de référence centrés sur la famille**<br>- Un accueil avec respect<br>- Un soin qui implique le mari et les accompagnants (communiquer avec promptitude, partager, solliciter, faire de la place, expliquer ce qu'on va faire a la femme)<br>- Donner de la place aux accompagnants depuis le début (CPN à post-partum) et leur accorder leur place de partenaires et aidants	Impliquer les hommes lors des CPN pour discuter ensemble du plan d'accouchement, ce qui peut leur permettre d'anticiper et de se préparer<br><br>Les maris sont des partenaires vivant l'expérience de la femme, ont le droit de participer et peuvent apporter aux soignants comme des partenaires pour surveiller, accompagner, convaincre et rassurer<br><br>Pour mesurer un soin centré sur la famille, tenir compte de ces points évoqués par les hommes et les femmes :<br>• Implication : Place accordée aux maris et aux accompagnants<br>• Communication prompte avec eux sans attendre qu'ils posent des questions<br>• Rapidite de la mise en œuvre de la référence et possibilité de payer après<br>• Une communication et des attitudes pour aider, soutenir la femme, mais aussi les maris et les accompagnants<br>• Être à l'écoute des besoins des femmes et faciliter leur satisfaction<br><br>NB : Les parents ont besoin qu'on reconnaisse, promeuve et facilite l'expression de leurs responsabilités, droits et compétences pour contribuer aux soins maternels et

SESSIONS	POINTS DE DISCUSSION	RESULTATS/RECOMMANDATIONS
	- Un soin qui donne la possibilité d'exprimer son droit et de se faire entendre<br>- Un soin qui discute le plan d'accouchement et le partage avec les maris et les accompagnants pour permettre d'anticiper et de se préparer	néonatal, tout en les considérant comme des partenaires et des collaborateurs et non ceux qui dérangentß
**Session 3**	**SOLIDARITE AVEC LES HOMMES ET LES FEMMES**<br>• Comprendre les **perspectives sur la signification et l'importance de l'intimité.**<br>• Tracer le **parcours de l'expérience de l'accouchement pour comprendre les moments où l'intimité serait appréciée.**	
	**Discussion autour de la notion de « Être ensemble, être solidaire »**<br>• *Signification de « togetherness » pour les parents*<br>- Être présent aux cotes de l'un et de l'autre<br>- Avoir la possibilité de suivre ce qui se passe quand on ne peut pas être présent physiquement<br>- Ne pas être séparé par l'information<br>- Rester connectés à leur épouse, à l'enfant par le biais de la sage-femme qui doit garder le lien avec eux tout au long de la prise en charge<br>• *Zéro séparation a été défini à plusieurs niveaux :*	Les parents sont conscients qu'en raison de la nature des soins d'accouchement, ils ne peuvent pas être présents physiquement pendant tout le processus, mais ils souhaitent qu'on leur reconnaisse :<br>- Leurs capacités à pouvoir se rendre utiles, d'aider<br>- Leurs droits à savoir ce qui se passe lorsqu'ils ne peuvent pas être présents physiquement<br>Les hommes sont de plus en plus intéressés à s'impliquer lors de l'accouchement. S'ils acceptent plus facilement

SESSIONS	POINTS DE DISCUSSION	RESULTATS/RECOMMANDATIONS
	- **Chez les hommes**<br>i. Entre le mari et sa femme parturiente : *se rassurer, devenir un acteur de l'accouchement, aider la sage-femme dans l'observance du traitement par la femme*<br>ii. Entre les accompagnants (de sexe féminin) et la femme parturiente<br>iii. Entre le mari et le nouveau-né : *pour se rassurer, emotions*<br>iv. Entre la femme parturiente, le bébé et la sage-femme : *besoin de quelqu'un pour surveiller et s'assurer que tout va bien*<br><br>- **Chez les femmes**<br>*i.* Entre la femme parturiente et son mari : *confiance, aide et soutien, communication rassurante*<br>*ii.* Entre la femme parturiente et le nouveau-né : *chacun d'eux a besoin de l'autre sur plan émotionnel et physique. Voir pour se rassurer*<br>*iii.* Entre la femme parturiente et la famille(en particulier les accompagnantes de sexe féminin) : *aider avec les habits, soutien pour préparer l'enfant, acteurs de la puériculture culturellement sensible (damp)*<br>iv. Entre la femme parturiente et la sage-femme : *surveiller et assister, donner les premiers conseils qui rassurent, se voir félicitée par elle*<br>v. Entre les parturientes (communautés d'expériences, d'apprentissage et de partage)	d'être séparés physiquement de leur femme lors du processus, la présence de la sage-femme est plus que requise lors de l'accouchement et du post-partum : celui à qui on délègue la responsabilité de surveiller, ne doit pas trop s'éloigner de ma femme et de mon enfant<br><br>Pour les parents, le moment clé qui nécessite de l'attention est l'arrivée : besoin de la présence de la sage-femme, qui offre un accueil personnalisé, avec une communication respectueuse et qui rassure, offre une aide<br><br>Le besoin d'un soin de soutien est fondamental pour les parents dès le moment de l'arrivée, avec de la considération et une attention pour les maris et les accompagnants<br><br>Les parents attendent de la sage-femme une présence et une attention, zéro-séparation avec la parturiente, avec

SESSIONS	POINTS DE DISCUSSION	RESULTATS/RECOMMANDATIONS
	**Cartographie du parcours**<br><br>• **Arrivée**<br>- Importance de l'accueil qui détermine la suite du processus : orientation, accompagnement et assistance<br>- Échographies et analyses, avec partage des résultats qui permettent à la femme et accompagnants d'en savoir un peu plus sur comment ça va se passer (d'où la nécessité de communiquer<br>- Disponibilité des médicaments est vécue comme une contrainte pour la continuité des soins<br>- Importance de zéro-séparation entre la sage-femme, la parturiente et les accompagnants<br><br>• **Travail :**<br>- Besoins en assistance<br>- Importance de zéro-séparation entre la sage-femme, la parturiente<br>- Informer les accompagnants pour les rassurer<br><br>• **Accouchement**<br>- Conditions d'hygiène importantes pour rassurer les femmes et les accompagnants<br>- Le personnel doit être prêt a intervenir et communiquer pour aider et rassurer<br>- Les équipements et le matériel doivent être disponibles et à portée de main	eux aux quatre moments de l'arrivée, du travail, de l'accouchement et du post-partum=<br>- gage de confiance, rassure les parents<br>- assure une surveillance étroite<br>- communication pour soutenir et conseiller<br><br>Les moments prioritaires de de zéro-séparation :<br>1. arrivée<br>2. accouchement<br>3. post-partum<br><br>L'intimité et la douleur, la patience que requiert le moment du travail est plus laissé a la discrétion et sous la seule responsabilité de la sage-femme

SESSIONS	POINTS DE DISCUSSION	RESULTATS/RECOMMANDATIONS
	- Avoir les hommes ou accompagnants pour les besoins urgents, achats de médicaments, assister la sage-femme, donner de la nourriture<br>- Importance de zéro-séparation entre la sage-femme et la parturiente ; entre la parturiente et les accompagnants<br>-<br>• **Post -partum**<br>- Conditions d'hygiène importantes pour rassurer les femmes et les accompagnants<br>- Présence et attention de la sage-femme pour la première tétée<br>- Félicitations a la parturiente sont motivantes<br>- Besoins de voir et toucher son enfant aussi bien pour la mère que le mari : se rassurer, communication<br>- Importance de la place laissée aux accompagnants de sexe féminin : soutien et aide<br>- Importance de zéro-séparation entre la sage-femme et la parturiente ; entre la parturiente et les accompagnants, entre le papa, la maman et le nouveau-né	
