# Supplementary material for: Using human-centered design to re-vision the emergency obstetric and newborn care framework: Insights from Bangladesh, Malawi and Senegal
Source: PLOS Glob Public Health. 2025 Jun 23;5(6):e0004771. doi: 10.1371/journal.pgph.0004771 (PMC12185017; doi:10.1371/journal.pgph.0004771)
Supplement: S2 Data — (PDF) [file pgph.0004771.s005.pdf]

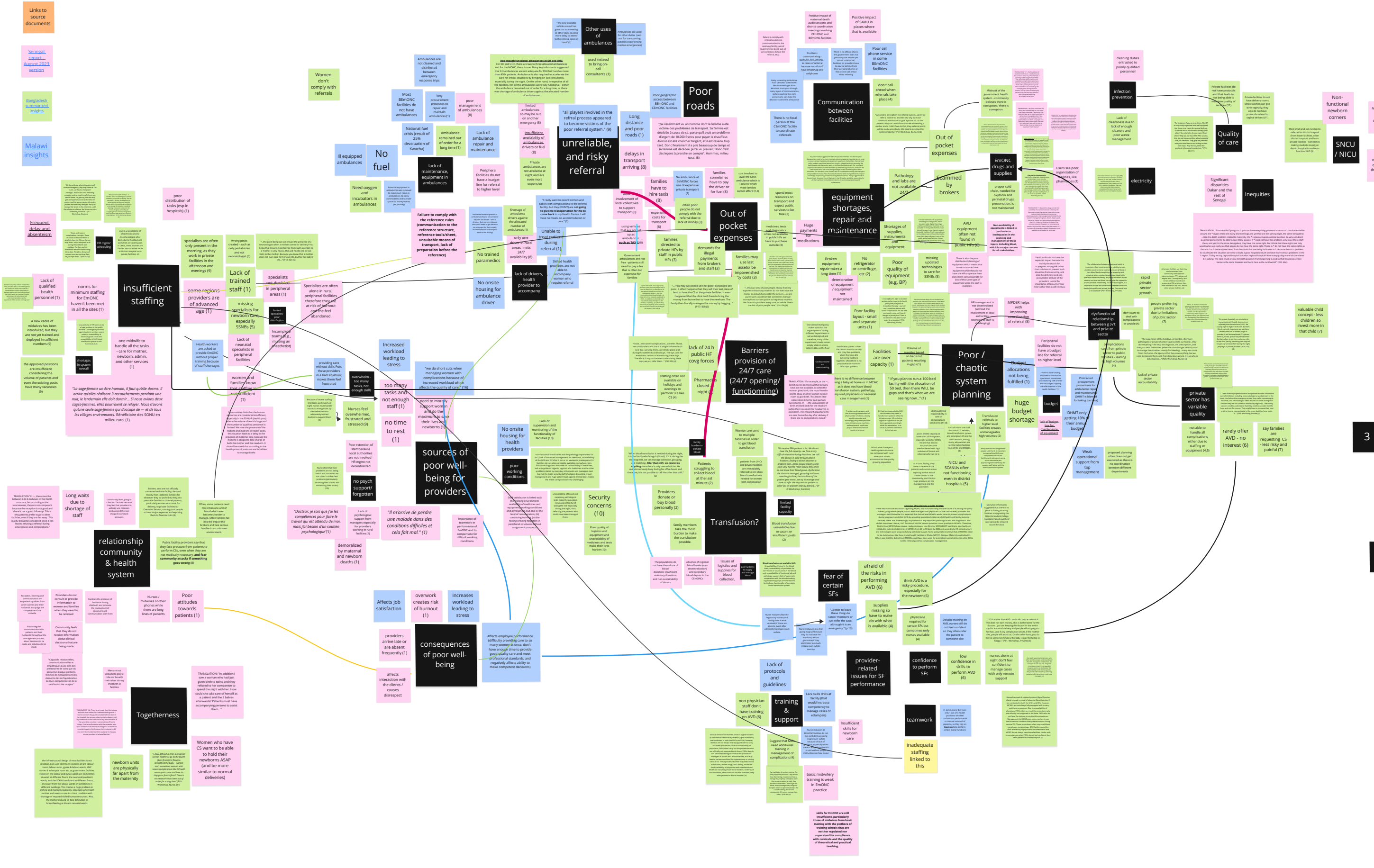

preliminary insights

how system affects relationship with community

1 System failures offload onto patient responsibility

e.g. payment for blood, go get labs or drugs elsewhere, find transport

Lack of strong national planning and coordination across system

Poor planning leads to overloaded facilities

under resourced health systems

2 Lack of planning passes responsibility to the front line workers,

3 referral / 24/7 gaps in coverage

Continuum of care disruption

shortage of staff, equipment

4 links between other system aspects and provider well being

how system affects relationship with community

5 Fear and lack of confidence affect SF performance

6 Care for women and care for newborns are not integrated

7 risky and dangerous referrals

New table

INSIGHT	BANGLADESH	MALAWI	SENEGAL
1 Women and newborns lives are in danger due to system failures caused by inadequate resources, planning and management<br>This is a meta-insight which underpins all of the other insights, and is more upstream than some of the others.	huge budget shortage<br>rapid private sector growth<br>lack of private sector accountability<br>proposed planning often done not yet executed as there is no coordination between different departments<br>Volume of supplies based on beds not patients resulting in gaps (1)<br>" if you plan to run a 100 bed facility with the allocation of 50 bed, then there WILL be gaps and that's what we are seeing now..." (1)<br>Selection of facilities to provide EmONC<br>[illegible]	There is little funding allocated to districts for operations, sometimes only receiving 10% of their annual budget crippling the effectiveness of the health facilities (1)<br>Budget allocations not being fulfilled (1)<br>Weak operational support from top management<br>DHMT only getting 10% of their annual budget	Budget partition issues: difference of opinion between providers and managers as to priorities<br>Peripheral facilities do not have a budget line for referral to higher level<br>lack of budget line for maintenance of equipment<br>Significant disparities Dakar and the rest of Senegal<br>[illegible]
2 Women and families struggle in emergencies to mobilize money and resources needed for medicines, blood transfusion and referral transport<br>OOP because of system shortages	Family members take on most of the burden to make blood transfusion possible,<br>Patients struggling to collect blood at the last minute (2)<br>demands for illegal payments from brokers and staff (3)<br>families directed to private HFs by staff in public HFs (3)<br>medicines, tests and diagnostics often not available in public HFs so have to purchase outside (3)<br>families may use last assets/ be impoverished by costs (3)<br>spend most resources on transport and expect public services to be free (3)<br>Scammed by brokers<br>[illegible]	No ambulance at BeMONC forces use of expensive private transport (1)	Huge payments for medications<br>using vehicles that are not set up as ambulances: such as TAXIS (8)<br>families sometimes have to pay the driver or for fuel (8)<br>expensive costs for transport (8)<br>[illegible]
3 Front line providers are faced with finding quick, often imperfect, solutions in chaotic environments, sometimes compromising quality of care<br>enabling environment/ readiness<br>Slightly changed wording: Health providers must find quick, often imperfect, solutions in chaotic under-resourced health facility environments, sometimes compromising quality of care	Providers donate or buy blood personally (2)<br>supplies missing so have to make do with what is available (4)<br>Shortages of supplies, instruments and equipment<br>AVD equipment often not found in public HFs (4)<br>No refrigerator or centrifuges etc (2)<br>Poor quality of equipment (4)<br>Lack of cleanliness due to lack of enough cleaners and poor management<br>missing updated technologies to care for SSNBs (5)<br>Facilities are over capacity (1)<br>Poor facility layout - small and separate units (1)<br>NICU and SCANUs often not functioning even in district hospitals (5)<br>[illegible]	Health workers are asked to provide EmONC without proper training because of staff shortages<br>"We do short cuts when managing women with complications because of increased workload which affects the quality of care." (p.16)<br>Affects employee performance (difficulty providing care to so many women at once, don't have enough time to provide good-quality care and meet professional standards, and negatively affects ability to make competent decisions)<br>[illegible]	poor distribution of tasks (esp in hospitals) (1)<br>Issues of logistics and supplies for blood collection<br>deterioration of equipment / equipment not maintained<br>No resuscitation equipment / resuscitation table not up to standards<br>Lack of oxygen and emergency drugs for the newborn<br>Users see poor organization of facilities, like pharmacies (1)<br>cleaning duties entrusted to poorly qualified personnel<br>[illegible]
4 Shortages of competent and skilled front line providers means not all women and newborns get quality care<br>adequate staffing<br>Changed slightly to: Shortages of competent health providers means not all women and newborns get good quality care	physicians required for certain SFs but sometimes only nurses available (4)<br>Lack of trained staff (1)<br>wrong posts created - such as only pediatrician and not neonatologist (5)<br>missing specialists for newborn care, especially SSNBs (5)<br>[illegible]	providers cannot manage all of the patients given shortages of staffing and have to make choices about who will receive what care<br>"We do short cuts when managing women with complications because of increased workload which affects the quality of care." (p.16)<br>Health workers are asked to provide EmONC without proper training because of staff shortages<br>[illegible]	"Midwives cannot care for both mother and newborn at the same time."<br>Frequent delays and absenteeism<br>specialists not doubled in peripheral areas (1)<br>one midwife to handle all the tasks - care for mother, newborn, admin, and other services (1)<br>norms for minimum staffing for EmONC haven't been met in all the sites (1)<br>Poor retention of staff because local authorities are not involved - HR mgmt not decentralized<br>Long waits due to shortage of staff<br>cleaning duties entrusted to poorly qualified personnel<br>[illegible]
5 Providers are afraid and lack confidence to perform certain EmONC signal functions due to poor support systems<br>supportive supervision, mentoring, medical education, etc.	afraid of the risks in performing AVD (6)<br>think AVD is a risky procedure, especially for the newborn (6)<br>non-physician staff don't have training on AVD (6)<br>nurses alone at night don't feel confident to manage cases with only remote support<br>physicians required for certain SFs but sometimes only nurses available (4)<br>supplies missing so have to make do with what is available (4)<br>[illegible]	Lack of protocols and guidelines<br>Despite training on AVD, nurses still do not feel confident so they often refer the patient to someone else<br>Lack skills drills at facility (that would increase competency to manage cases of eclampsia)<br>Health workers are asked to provide EmONC without proper training because of staff shortages<br>"...better to leave these things to senior members or just refer the case, although it is an emergency." (p.13)<br>[illegible]	basic midwifery training is weak in EmONC practice<br>Insufficient skills for newborn care<br>[illegible]
6 Providers well-being is compromised due to poor support from the system and strained relationships with communities	Public facility providers say that they face pressure from patients to perform CS, even when they are not medically necessary, and fear community attacks if something goes wrong (8)<br>providers have security concerns and fear attacks from communities when things go wrong<br>Nurses feel overwhelmed, frustrated and stressed (9)<br>Nurses feel that their problems are not being heard and initiatives are not taken to solve their problems within the system (particularly lessening their duties and addressing their stress)<br>Security concerns (10)<br>Poor quality of logistics and equipment and unavailability of medicines and tests make their tasks harder (10)<br>[illegible]	Providing care without the necessary skills and sufficient staffing leads to staff feeling frustrated and stressed<br>lack of onsite housing adds to this<br>Increased workload leading to stress<br>Affects job satisfaction<br>"we do short cuts when managing women with complications because of increased workload which affects the quality of care." (p.16)	Lack of psychological support from managers especially for providers working in rural facilities (1)<br>"Doctor, I know that I have the competencies to do the work expected of me, but I have a need of psychological support." (1)<br>overwork creates risk of burnout (1)<br>providers arrive late or are absent frequently (1)<br>affects interaction with the clients / causes disrespect<br>too many tasks and not enough staff (1)<br>no time to rest (1)<br>"Il m'arrive de perdre une malade dans des conditions difficiles et cela fait mal." (1)<br>demoralized by maternal and newborn deaths (1)<br>need to morally support women and do the maximum to save their lives and newborns (1)<br>Providers do not communicate with women and families and do not show empathy towards them, leading to conflicting relationships between them.<br>"Capacités relationnelles, communicationnelles et empathiques aussi bien des prestataires de corps que du personnel d'appui (gardiens, femmes de ménages) sont des éléments clés de l'appréciation de leurs compétences et de la satisfaction des usagers"<br>Most of the women who come to this hospital have a Cesarean section and I cannot tell you why. It really intrigues me. I don't know if it's wrong or what, but really I don't understand<br>[illegible]
7 Women's and newborns' continuous care is disrupted due to poor system readiness<br>excessive referrals, eg due to lack of readiness at sending facility<br>Slightly reworded this, for Continuous care for women and newborns is disrupted due to poor system readiness	lack of 24 h public HF covg forces<br>Pharmacies closed at night (1)<br>Pathology and labs are not available 24/7<br>staffing often not available on holidays and evenings to perform SFs like CS (4)<br>[illegible]	"...better to leave these things to senior members or just refer the case, although it is an emergency." (p.13)<br>Despite training on AVD, nurses still do not feel confident so they often refer the patient to someone else	"...at the Health Centre, beneficiaries pointed out that beds are not available, so when a woman gives birth, the must free the bed to allow another woman to have room to give birth. This leaves little observation time for post partum surveillance."
8 Systems fail newborns due to lack of focus on the dyad and limited integration of maternal and newborn services	SCANU are often not functional<br>NICU and SCANUs often not functioning even in district hospitals (5)<br>newborn units are physically far apart from the maternity<br>missing specialists for newborn care, especially SSNBs (5)<br>missing updated technologies to care for SSNBs (5)<br>[illegible]		Providers feel that there has not been enough emphasis on saving the newborns<br>Insufficient skills for newborn care<br>Non-functional newborn corners<br>No resuscitation equipment / resuscitation table not up to standards<br>Lack of oxygen and emergency drugs for the newborn<br>[illegible]
9 Referral system weaknesses pose mental and physical danger to women, babies, providers and drivers	ambulances not fully functional or fully equipped for complications<br>Need oxygen and incubators in ambulances<br>Ambulance remained out of order for a long time (1)<br>ambulances used instead to bring on-call consultants (1)<br>Private ambulances are not available at night and are even more expensive<br>No trained paramedics<br>Shortage of ambulance drivers against the allocated number of ambulances (1)<br>don't call ahead when referrals take place (4)<br>"we need to strengthen the referral system...when we refer a mother to another DH, why don't we communicate that DH or give a phone no. to the patient? Why can't we inform that we are sending a mother and a child? If we do that, they (referral point) will be ready accordingly. We need to develop this system instantly." (4)<br>[illegible]	"all players involved in the refrral process appeared to become victims of the poor referral system." (p.9)<br>Unable to treat patient during referral (1)<br>Most BEmONC facilities do not have ambulances<br>Long distance and poor roads (1)<br>Ill equipped ambulances<br>No fuel<br>Lack of ambulance repair and maintenance<br>long procurement processes to repair ambulances<br>No onsite housing for ambulance driver<br>National fuel crisis (result of 25% devaluation of Kwacha)<br>[illegible]	Sometimes have to use a vehicle that is not set up as an ambulance, such as taxis,<br>have only one driver in rural areas for ambulances so not available 24/7<br>ambulances are not well maintained<br>delays in transport arriving (8)<br>Failure to comply with the referral guidelines<br>Peripheral facilities do not have a budget line for referral to higher level<br>families have to hire taxis (8)<br>poor management of ambulances (8)<br>limited ambulances so may be out on another emergency (8)<br>No ambulances / insufficient availability of ambulances / drivers or fuel (8)<br>weak involvement of local collectives to support transport (8)<br>using vehicles that are not set up as ambulances: such as TAXIS (8)<br>families sometimes have to pay the driver or for fuel (8)<br>expensive costs for transport (8)<br>[illegible]
